# Tri-Culture Fermentation of Neem (*Azadirachta indica*) Leaves Induces Phytochemical Remodeling and Enhances Multi-Target Bioactivities Relevant to Androgenetic Alopecia

**DOI:** 10.3390/plants15182779

**Published:** 2026-09-10

**Authors:** Anurak Muangsanguan, Niphawan Panti, Warintorn Ruksiriwanich, Kasirawat Sawangrat, Pattarapa Pummara, Pornchai Rachtanapun, Korawan Sringarm, Sarana Rose Sommano, Sucheewin Krobthong, Chaiwat Arjin, Apinya Satsook, Yodying Yingchutrakul, Juan Manuel Castagnini

**Affiliations:** 1 Department of Pharmaceutical Sciences, Faculty of Pharmacy, Chiang Mai University, Chiang Mai 50200, Thailand; anurak_m@cmu.ac.th (A.M.); warintorn.ruksiri@cmu.ac.th (W.R.); kasirawat.s@cmu.ac.th (K.S.); 2 Faculty of Agro-Industry, Chiang Mai University, Chiang Mai 50100, Thailand; pattarapa_pummara@cmu.ac.th (P.P.); pornchai.r@cmu.ac.th (P.R.); 3 Cluster of Agro Bio-Circular-Green Industry (Agro BCG), Chiang Mai University, Chiang Mai 50200, Thailand; korawan.s@cmu.ac.th (K.S.); sarana.s@cmu.ac.th (S.R.S.); 4 Cluster of Valorization and Bio-Green Transformation for Translation Research Innovation of Raw Materials and Products, Chiang Mai University, Chiang Mai 50200, Thailand; 5 Department of Animal and Aquatic Sciences, Faculty of Agriculture, Chiang Mai University, Chiang Mai 50200, Thailand; chaiwat.arjin@cmu.ac.th; 6 Department of Plant and Soil Sciences, Faculty of Agriculture, Chiang Mai University, Chiang Mai 50200, Thailand; 7 Center of Excellence in Natural Products Chemistry (CENP), Department of Chemistry, Faculty of Science, Chulalongkorn University, Bangkok 10330, Thailand; sucheewin.k@chula.ac.th; 8 Office of Research Administration, Chiang Mai University, Chiang Mai 50200, Thailand; apinya.satsook@cmu.ac.th; 9 National Center for Genetic Engineering and Biotechnology, National Science and Technology Development Agency, Pathum Thani 12120, Thailand; yodying.yin@biotec.or.th; 10 Research Group in Innovative Technologies for Sustainable Food (ALISOST), Department of Preventive Medicine and Public Health, Food Science, Toxicology and Forensic Medicine, Faculty of Pharmacy, Universitat de València, Avenida Vicent Andrés Estellés s/n, 46100 Burjassot, Spain; juan.castagnini@uv.es

**Keywords:** 5α-reductase inhibition, androgenetic alopecia, *Azadirachta indica*, hair growth-promoting pathway, microbial fermentation

## Abstract

Androgenetic alopecia (AGA) involves dihydrotestosterone-driven follicular miniaturization compounded by oxidative stress and perifollicular inflammation, while current pharmacotherapies remain limited by adverse effects. Neem (*Azadirachta indica* A. Juss., Meliaceae) leaves, a phenolic- and limonoid-rich plant widely used in Southeast Asian traditional medicine, were fermented for seven days with a tri-culture consortium of *Saccharomyces cerevisiae*, *Lactobacillus plantarum*, and *Aspergillus niger* (TRI-NE) to evaluate whether expanded microbial diversity enhances bioactivity relative to the unfermented extract (UN-NE). TRI-NE significantly increased total phenolic content, rising from 315.30 to 564.70 mg GAE/g by day 7, alongside an overall enhancement of antioxidant capacity across all assays. Moreover, untargeted metabolomics revealed compositional remodeling, with 67.47% of significantly altered metabolite features upregulated after fermentation, including selective enrichment of quercetin despite reductions in other polyphenols. In human hair follicle dermal papilla cells (HFDPCs) and complementary models, TRI-NE consistently outperformed UN-NE, enhancing paracrine-mediated fibroblast proliferation, preserving cell viability under potassium-channel blockade, suppressing lipopolysaccharide-induced inflammatory nitric oxide production, and attenuating oxidative membrane damage. At the transcriptional level, TRI-NE downregulated androgen metabolism (*SRD5A1* and *SRD5A2*) and pro-regression genes (*TGFB1*) while upregulating Wnt/β-catenin (*CTNNB1*), Sonic Hedgehog (*SHH*, *SMO*, and *GLI1*), and angiogenic (*VEGF*) pathway genes, with effects matching or exceeding standard hair-loss therapeutics. These findings indicate that TRI-NE confers superior bioactivity over the unfermented extract, supporting its potential as a multi-target cosmeceutical candidate for AGA.

## 1. Introduction

The global prevalence of androgenetic alopecia (AGA) continues to expand alongside demand for effective, well-tolerated treatment options, yet the pharmacological standard of care has remained essentially unchanged for decades [1]. Finasteride and dutasteride, both 5α-reductase inhibitors, carry a recognized burden of sexual and endocrine adverse effects that limits sustained adherence, while minoxidil produces benefits that are largely reversible on cessation and do not address the inflammatory or oxidative components of follicular regression [2,3]. This persistent gap has sustained interest in plant-derived, multi-target alternatives, but it has also surfaced a recurring limitation of raw botanical extracts: their therapeutic ceiling is often set less by the phytochemical richness of the source material than by how much of that content the plant matrix makes accessible in a bioavailable form [4].

Microbial fermentation has become one of the more direct strategies for closing this accessibility gap. Fermenting microorganisms supply enzymatic machinery that plants themselves lack, including glycosidases, esterases, and phenolic acid decarboxylases capable of releasing phenolics and terpenoids from their glycosylated or esterified forms, while simultaneously generating classes of bioactive metabolites, such as organic acids, peptides, and exopolysaccharides, which are absent from the unfermented tissue altogether [5,6,7]. However, the optimal degree of microbial diversity within a fermenting consortium required to maximize these benefits remains underexplored, particularly regarding whether synergistic effects scale linearly with the addition of functionally distinct organisms.

In our previous study, mixed-culture fermentation of coffee pulp using a consortium of *Saccharomyces cerevisiae* and *Lactobacillus plantarum* significantly enhanced metabolomic diversity and biological activities relevant to AGA relative to the corresponding controls. Building on this fermentation strategy, the present study incorporated *Aspergillus niger* as a third microorganism, based on its reported ability to promote phytochemical transformation and enhance phenolic recovery and antioxidant activity during solid-state fermentation of plant matrices [8], and applied the resulting tri-culture consortium to neem leaves. The present study was therefore designed to characterize the phytochemical remodeling and AGA-relevant biological activities associated with tri-culture fermentation in a chemically distinct botanical substrate. Because a corresponding dual-culture neem treatment was not included, the present experimental design does not permit direct determination of the incremental contribution of *A. niger* relative to the *S. cerevisiae*–*L. plantarum* consortium.

The three organisms were selected based on complementary functions reported in previous fermentation studies. *S. cerevisiae* contributes saccharolytic activity together with yeast-derived metabolites such as β-glucans [9]. *L. plantarum* possesses enzymatic activities capable of modifying phenolic conjugates and has been shown to induce substantial changes in the phenolic profile of fermented plant materials [10]. *A. niger* has been widely applied in solid-state fermentation of plant matrices and has been reported to enhance the recovery and transformation of phenolic and flavonoid compounds, accompanied by increased antioxidant activity [8]. These literature-reported properties provided the rationale for incorporating *A. niger* into the consortium with the expectation of broadening the potential biotransformation capacity of the fermentation system. However, the specific contribution of *A. niger* to the effects observed in TRI-NE cannot be distinguished from those of the other consortium members in the present experimental design.

To evaluate the potential of this consortium on a novel plant-based substrate, neem (*Azadirachta indica* A. Juss., Meliaceae) leaves were selected. Neem is a naturalized species throughout Thailand and mainland Southeast Asia, widely consumed as a regular household vegetable and established as a culturally embedded medicinal plant in the region [11,12]. Beyond this local familiarity, the tree has an extensive documented pharmacology: its limonoid- and flavonoid-rich chemistry, including azadirachtin, nimbolide, nimbin, gedunin, quercetin, gallic acid, and catechins, underlies well-documented antioxidant and anti-inflammatory activities, together with additional antimicrobial and immuno-modulatory effects reported across the literature [12].

Neem was chosen for this comparison specifically because its two dominant bioactivity classes, antioxidant and anti-inflammatory, intersect AGA pathophysiology at points where a fermentation-driven bioavailability gain should be mechanistically detectable. Follicular regression in AGA is not purely hormonal: dihydrotestosterone (DHT) engagement of the androgen receptor in human hair follicle dermal papilla cells (HFDPCs) drives TGF-β1-mediated apoptotic signaling that accelerates the transition from the growth phase (anagen) to the regression phase (catagen)—the first two of the four phases comprising the hair cycle, followed by the resting phase (telogen) and the shedding phase (exogen)—while the accompanying rise in reactive oxygen species produces lipid peroxidation that further compromises HFDPCs function; together these converge on suppression of the Wnt/β-catenin-Sonic Hedgehog axis and VEGF-dependent angiogenesis that the follicle otherwise depends on to remain in the growth phase (anagen) [2,13]. HFDPCs additionally act as paracrine regulators of the perifollicular niche, and disruption of HFDPC-fibroblast signaling under androgen excess and oxidative stress is increasingly recognized as a contributor to follicular regression in its own right [2]. Because neem already exhibits antioxidant and anti-inflammatory activities in its unfermented state, it provides an appropriate substrate for evaluating whether tri-culture fermentation confers additional biological benefits beyond its intrinsic pharmacological activity. Any enhanced modulation of androgen metabolism, paracrine signaling, or hair-growth pathways would therefore be attributable primarily to fermentation-induced compositional changes rather than to the inherent properties of the plant itself.

In this study, neem was fermented for seven days with the *S. cerevisiae*, *L. plantarum*, and *A. niger* consortium (tri-culture-fermented, TRI-NE) and compared with the unfermented extract (UN-NE) through two complementary analyses: (i) phytochemical and untargeted metabolomic characterization of fermentation-induced compositional changes and (ii) mechanistic evaluation of the associated biological activities, spanning HFDPC viability and proliferation, HFDPC-to-fibroblast paracrine signaling, ATP-sensitive potassium (K_ATP_) channel-dependent HFDPC viability, lipopolysaccharide (LPS)-induced inflammatory nitric oxide production, and oxidative stress (intracellular ROS and lipid peroxidation), as well as the expression of genes associated with hair loss, including androgen metabolism (*SRD5A1* and *SRD5A2*), transforming growth factor beta 1 (*TGFB1*)-mediated follicular regression, and hair growth pathways, including the Wnt/β-catenin (*CTNNB1*), Sonic Hedgehog (*SHH*, *SMO*, and *GLI1*), and angiogenesis (*VEGF*) growth axis. Ultimately, this approach aims to evaluate the capacity of a rationally designed tri-culture fermentation system to enhance the therapeutic potential of neem leaves while providing mechanistic insight into how fermentation-induced compositional changes translate into improved biological outcomes for AGA management.

## 2. Results

### 2.1. Phytochemical Characteristics and Antioxidant Properties of Neem Extracts

Neem-derived extracts were prepared for comparative evaluation in this study: an unfermented crude extract (UN-NE) and an extract obtained after tri-culture fermentation using a 1:1:1 combination of *S. cerevisiae*, *L. plantarum*, and *A. niger* (TRI-NE). Both extracts were recovered using ultrasonic-assisted extraction with 95% (*v*/*v*) ethanol as the extraction solvent. Both concentrated extracts appeared as dark greenish-brown liquids and exhibited broadly similar visual characteristics. However, TRI-NE showed a slightly more pronounced brown hue than UN-NE. As this observation was qualitative, the subtle color difference may reflect fermentation-associated changes in pigments, phenolic constituents, or other metabolites generated during microbial fermentation [14].

Extraction yield was additionally estimated using the volumetric–gravimetric dry-residue approach described in Section 3.2.4. Across the 7-day incubation period, the extraction yield ranged from 9.74 to 10.56% (*w*/*w*) for UN-NE and from 9.58 to 10.42% (*w*/*w*) for TRI-NE.

**Figure 1:**
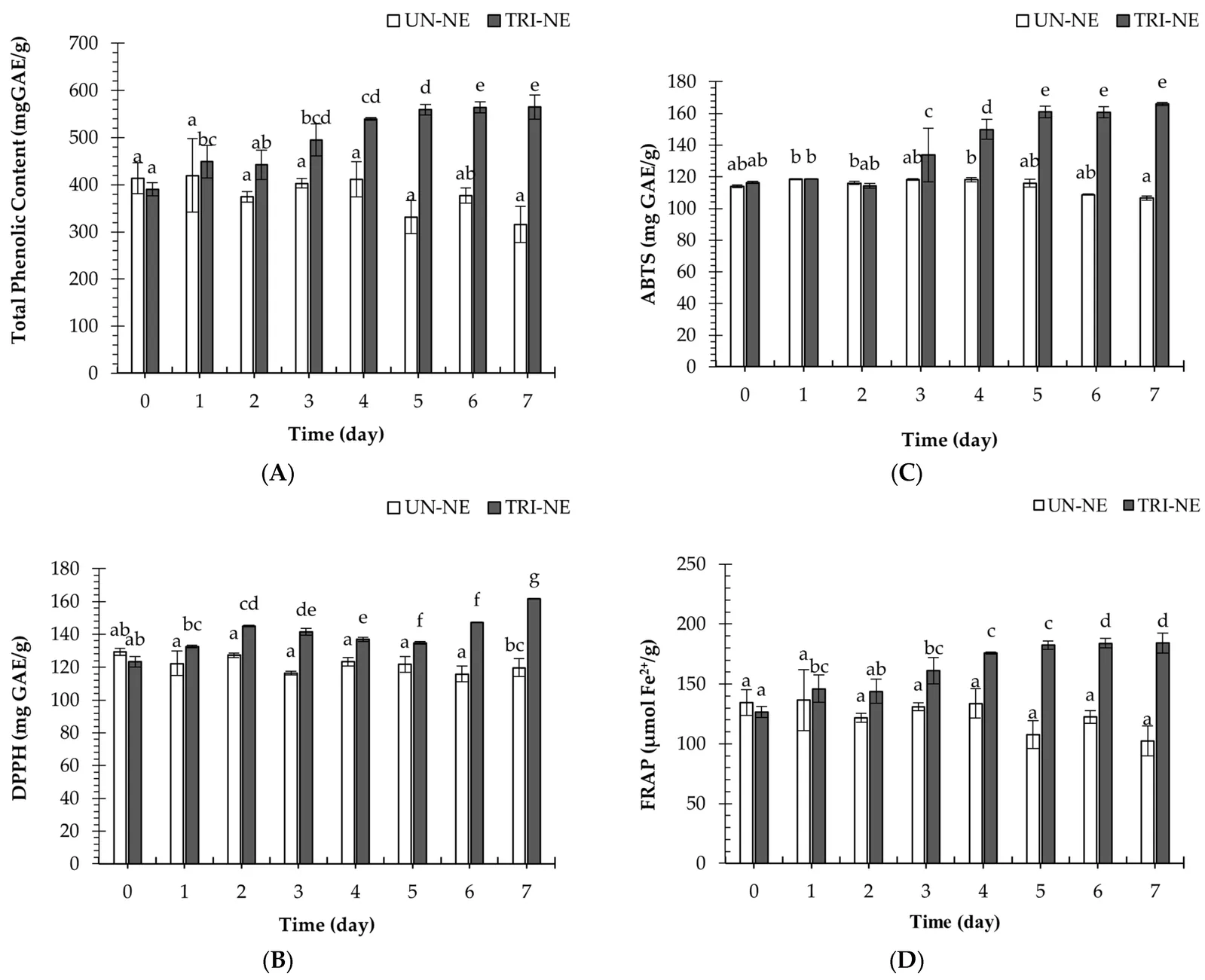
Effect of fermentation time on total phenolic content (TPC) (**A**), DPPH radical scavenging activity (**B**), ABTS radical scavenging activity (**C**), and ferric reducing antioxidant power (FRAP) (**D**) of fermented neem extracts during fermentation at 30 °C. Lowercase letters represent significant differences between treatment groups over time (*p* < 0.05). Unfermented neem, UN-NE; tri-culture (*Saccharomyces cerevisiae*, *Lactobacillus plantarum*, and *Aspergillus niger*) fermented neem, TRI-NE. Data are presented as mean ± SD (*n* = 3).

As shown in Figure 1A, total phenolic content (TPC) of neem extracts was significantly influenced by fermentation and incubation time. In UN-NE, TPC showed no consistent time-dependent trend during the 7-day incubation period, suggesting that without active microbial fermentation, substantial liberation of bound phenolics does not occur over this period. In contrast, fermentation with a 1:1:1 mixed culture of *S. cerevisiae*, *L. plantarum*, and *A. niger* significantly enhanced TPC in TRI-NE over time, with the most pronounced increases occurring from day 1 onward. These values represent substantial increases over the day-0 fermentation baseline and the matched UN-NE control. The increase in TPC may reflect the combined hydrolytic and metabolic activities of the tri-culture consortium. Previous studies have demonstrated that *A. niger*-mediated solid-state fermentation can enhance the release and transformation of phenolic constituents in plant matrices [12,15,16,17], while *L. plantarum* can modify phenolic profiles through microbial enzymatic biotransformation [11,18,19,20]. However, because mono- and dual-culture controls and direct measurements of strain-specific enzymatic activities were not included, the relative contribution of each microorganism to the observed increase in TPC cannot be determined from the present data.

Consistent with the TPC results, 2,2-diphenyl-1-picrylhydrazyl (DPPH) radical scavenging activity of neem differed significantly between UN-NE and TRI-NE and across fermentation time. In UN-NE, DPPH activity did not exhibit a clear time-dependent progression across the 7-day period. TRI-NE showed significantly higher DPPH values compared to UN-NE, with time-dependent increases marked by ascending lowercase letters. The highest DPPH value at day 7 in TRI-NE was 161.78 mg GAE/g (Figure 1B). The fermentation-induced enhancement of DPPH activity may be associated with microbial hydrolysis and biotransformation of complex or conjugated phenolic compounds into lower-molecular-weight forms with altered radical-scavenging properties [18,19,21] Such mechanisms are consistent with previous fermentation studies; however, the specific enzymes and individual microorganisms responsible for the observed changes were not directly determined in the present study.

A similar trend was observed for 2,2′-azino-bis(3-ethylbenzothiazoline-6-sulfonic acid) (ABTS) radical scavenging activity, which was significantly affected by treatment (UN-NE vs. TRI-NE) and time. In UN-NE, ABTS activity showed the absence of a strong progressive trend. TRI-NE demonstrated substantially elevated ABTS activity relative to UN-NE at matched time points, with statistically significant increases becoming most prominent from day 3 onward (Figure 1C). By day 7, TRI-NE reached 165.94 mg GAE/g, substantially higher than the corresponding UN-NE value. The marked increase in ABTS activity beyond day 3 may similarly reflect progressive microbial transformation during fermentation. Oxidative and hydrolytic activities reported during fungal fermentation, together with low-molecular-weight metabolites generated during lactic acid fermentation, may contribute to enhanced ABTS-measurable antioxidant activity [20,22,23].

In line with these findings, the ferric reducing antioxidant power (FRAP) of neem showed significant differences between UN-NE and TRI-NE and over time. In UN-NE, FRAP values showed minimal statistically significant temporal change, confirming that spontaneous liberation of reducing phenolics is negligible over 7 days without microbial activity, and the substrate phenolic composition remains largely stable during incubation at 30 °C alone. TRI-NE exhibited markedly and progressively elevated FRAP values, reaching 183.96 µmol Fe^2+^/g extract by day 7 (Figure 1D). These gains are attributed to the accumulation of structurally simple, redox-active phenolics such as gallic acid, catechol, and pyrogallol derivatives, generated through microbial hydrolysis and decarboxylation of complex phenolic polymers during the later stages of fermentation [24,25,26].

### 2.2. Global Metabolomic Profiling and Multivariate Statistical Analysis of Fermented Neem Extract

A total of 2327 annotated metabolite features were retained for downstream comparative analysis between the fermented sample and the non-fermented control. In the overall dataset without statistical cutoff, 61.19% of metabolites showed increased abundance in the fermented sample relative to the control, while 38.76% exhibited decreased abundance. This indicates a general trend toward metabolite accumulation following fermentation. When applying a stringent significance filter (adjusted *p*-value < 0.01 and fold change > 2 or <0.5), the number of significantly altered metabolites decreased. Among these, 67.47% of metabolites were significantly up-regulated, whereas 32.53% were down-regulated.

**Figure 2:**
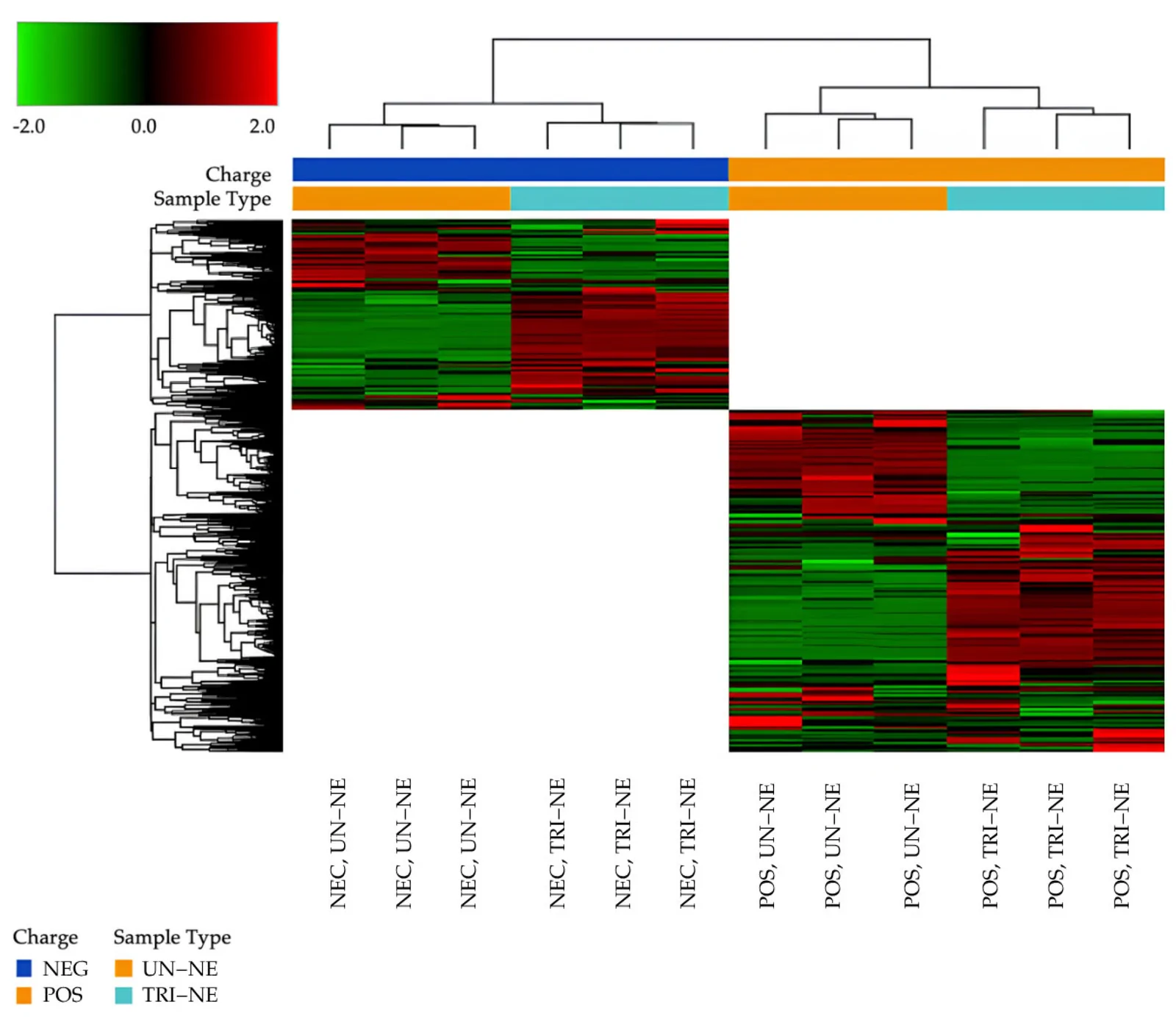
Heatmap and hierarchical clustering of putatively annotated metabolites in fermented samples and non-fermented controls. The analysis was performed using Euclidean distance and complete linkage clustering on scaled data. Each row represents a metabolite, and each column represents a sample. Red indicates higher relative abundance, while green indicates lower relative abundance. Samples are grouped by ionization mode (POS and NEG) and sample type (control and fermented). The clear separation between control and fermented samples highlights distinct metabolic profiles and demonstrates the impact of fermentation on metabolite composition.

Hierarchical clustering analysis further revealed a clear separation between fermented samples and non-fermented controls, demonstrating distinct metabolic profiles between the two groups (Figure 2). The clustering pattern was consistent across both ionization modes (positive and negative), indicating good reproducibility within groups and substantial metabolic divergence induced by fermentation. These results indicate that fermentation induces a pronounced metabolic shift, predominantly characterized by an increase in metabolite abundance relative to the non-fermented control.

### 2.3. Polyphenol Profile of Neem Extracts

The polyphenol composition of the neem extracts was determined via HPLC analysis (Table 1). Overall, UN-NE contained higher levels of individual polyphenol compounds compared to TRI-NE. Specifically, the concentrations of chlorogenic acid (15.85 ± 0.12 and 10.86 ± 0.48 mg/g extract), catechin (18.55 ± 0.22 and 16.59 ± 0.25 mg/g extract), epicatechin (4.06 ± 0.11 and 2.27 ± 0.01 mg/g extract), and *p*-coumaric acid (2.84 ± 0.03 and 0.10 ± 0.03 mg/g extract) were significantly higher in UN-NE than in TRI-NE, respectively. Additionally, naringin was detected exclusively in UN-NE but was not detected in TRI-NE, whereas gallic acid and *o*-coumaric acid contents remained comparable between the two extracts.

In contrast, the quercetin content showed a significant increase from 10.23 ± 0.75 mg/g extract in UN-NE to 15.39 ± 1.97 mg/g in TRI-NE. This enhancement is likely attributable to microbial enzymatic hydrolysis of quercetin glycosides, resulting in the liberation of the aglycone form [10]. Quercetin is widely recognized for its potent antioxidant and anti-inflammatory properties [27]. Notably, it has also been reported to inhibit SRD5A activity in HFDPCs, a key enzyme implicated in the pathogenesis of AGA [28]. This chemical discrepancy highlights the broader impact of the tri-culture fermentation process, which dynamically reshaped the global chemical landscape of the extract. Beyond the selective enrichment of quercetin observed by targeted HPLC analysis, untargeted metabolomic profiling revealed that fermentation induced broader compositional remodeling across multiple metabolite groups. Among the significantly upregulated features were putatively annotated flavonoid and related metabolites. Several phenolic acids and related compounds, including isoferulic acid, salicylic acid, and vanillic acid, were also significantly increased following fermentation. In addition, fatty acid- and lipid-related metabolites, including linoleic acid, oleic acid, and the oxylipin-related metabolite 12(13)-EpOME, were enriched in TRI-NE. These findings indicate that the enhanced biological properties of TRI-NE are unlikely to be attributable to quercetin alone but may instead reflect the collective contribution of multiple fermentation-derived or biotransformed metabolites with diverse chemical characteristics. The complete list of significantly altered metabolite features, together with their mass-to-charge ratios, retention times, fold changes, and adjusted *p*-values, is provided in Supplementary Table S4. Nevertheless, because metabolite assignments were derived from untargeted LC–MS/MS analysis, these annotations should be regarded as putative and warrant further targeted confirmation.

### 2.4. Cell Culture and Cytotoxicity Assessment

Cytotoxicity of UN-NE and TRI-NE was evaluated across a ten-point dilution series (0.004–2.000 mg/mL) in HFDPCs, hTERT fibroblasts, RAW 264.7, and DU-145 cells, in accordance with ISO 10993-5, which defines ≥80% viability as the threshold for non-cytotoxicity [29]. RAW 264.7 and DU-145 cells tolerated both extracts up to 0.125 mg/mL, whereas HFDPC and hTERT fibroblast viability fell below the acceptable threshold beyond 0.0625 mg/mL. Accordingly, 0.0625 mg/mL was defined as the highest concentration that remained non-cytotoxic across all cell types and both extracts and was therefore selected as the working concentration for all downstream assays.

The four cell types were selected for their complementary biological roles, with each serving a distinct experimental purpose rather than acting as interchangeable models. DU-145 cells, despite originating from prostatic tissue rather than the hair follicle, constitutively express high levels of 5α-reductase and are therefore routinely used to cross-validate androgen-metabolism findings obtained in HFDPCs [29,30]. As the primary functional component of the hair bulb, the proliferation of HFDPCs directly governs the hair follicle cycle and drives hair growth [31]. RAW 264.7 macrophages served as a complementary model for validating inflammatory responses owing to their well-characterized, robust, and reproducible response to lipopolysaccharide stimulation, thereby providing mechanistic support for the findings obtained in HFDPCs [32,33]. Lastly, hTERT fibroblasts were included as the responder population for conditioned-medium experiments to evaluate indirect biological effects mediated by HFDPC-secreted paracrine factors within the follicular microenvironment [34].

Notably, a direct comparison between UN-NE and TRI-NE at each tested concentration revealed no shift in the toxicity ceiling attributable to fermentation; both extracts exhibited comparable cytotoxicity profiles across the cell panel. This confirms that enzymatic biotransformation did not compress the safety window relative to the unfermented substrate, thereby ensuring that subsequent functional differences reflect compositional enhancements rather than altered cytotoxic burdens. Consequently, 0.0625 mg/mL was established as the baseline concentration to evaluate both extracts on an equitable safety footing. This framework defines and rationalizes the specific cellular pairings carried forward: HFDPCs and DU-145 for androgenic endpoints, RAW 264.7 alongside HFDPCs for inflammatory readouts, and hTERT fibroblasts as effectors for paracrine signaling. The cell viability profiles across the entire concentration range for all evaluated cell lines are compiled in Supplementary Table S1.

### 2.5. Paracrine-Mediated Fibroblast Proliferation by Neem Extracts

**Figure 3:**
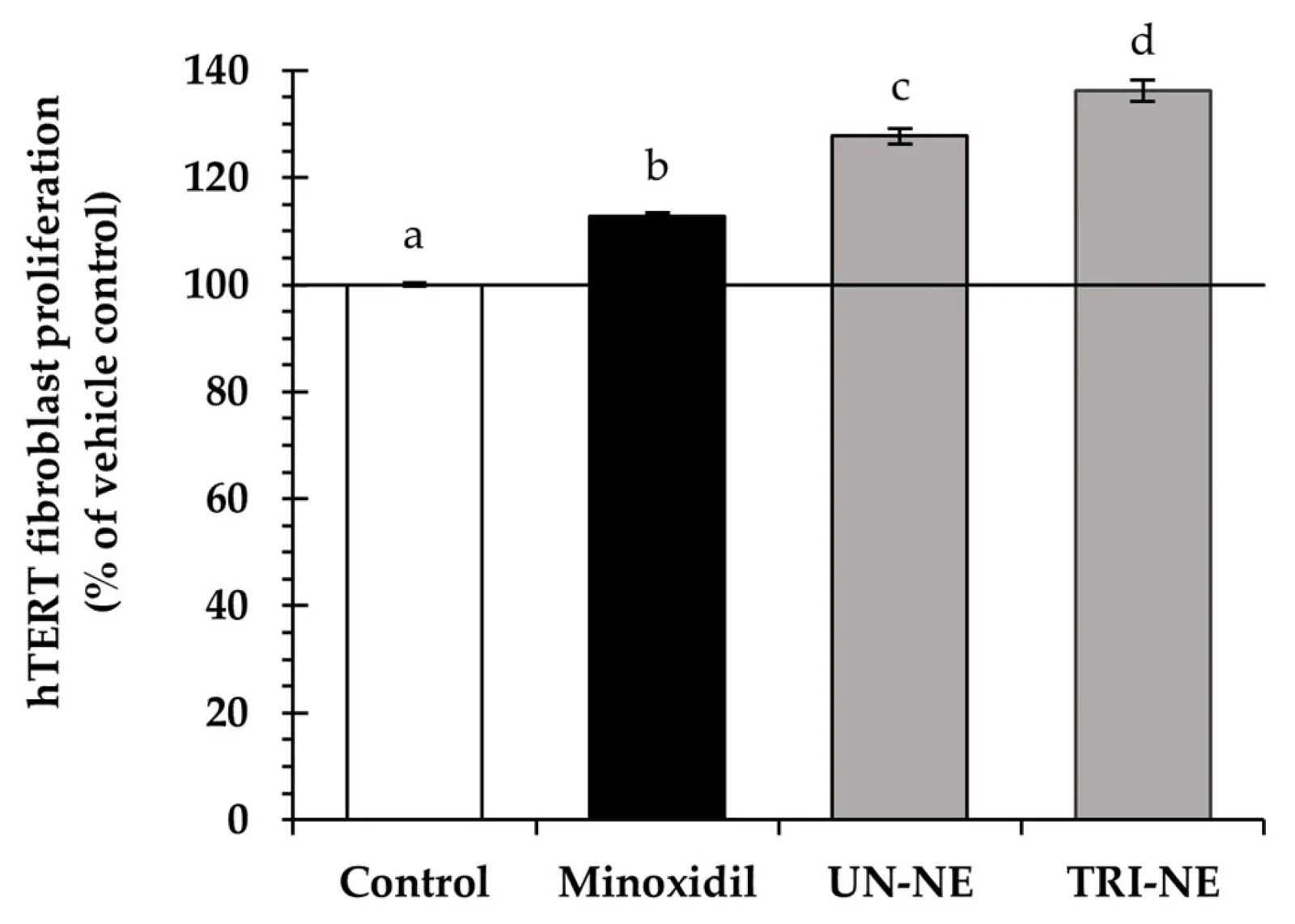
Paracrine-mediated proliferative effects of hTERT fibroblasts cultured in conditioned medium collected from HFDPCs pre-treated with tri-culture fermented neem extract (TRI-NE), unfermented neem extract (UN-NE), or a standard treatment (minoxidil) at 0.0625 mg/mL, relative to the vehicle control (conditioned medium from untreated HFDPCs). Values are expressed as mean ± SD (*n* = 3). One-way ANOVA followed by Tukey’s HSD post hoc test was applied; groups sharing no common letter (a–d) differ significantly (*p* < 0.05).

Perifollicular fibroblasts are structurally crucial to the dermal niche. However, their proliferation and function largely rely on paracrine signals and growth factors secreted by HFDPCs rather than responding directly to systemic or oxidative stimuli [35]. To evaluate this intercellular signaling axis, a conditioned-medium model was utilized. In our study, HFDPCs were exposed to UN-NE, TRI-NE, or minoxidil at a concentration of 0.0625 mg/mL, and the resulting supernatants were subsequently transferred to hTERT fibroblast cultures (Figure 3). The TRI-NE-conditioned medium achieved the highest rate of fibroblast proliferation (136.17 ± 1.48% of the control), outperforming both UN-NE (127.69 ± 1.93% of the control) and the positive control minoxidil (112.74 ± 0.65%), with all treatment groups exhibiting statistically significant increases relative to the untreated baseline.

The biological activity of this study correlates strongly with the bioactive constituents altered during the fermentation process, particularly quercetin. Quantitative HPLC analysis revealed that while several polyphenol compounds declined post-fermentation, the quercetin content within the TRI-NE extract increased significantly, rising from 10.23 ± 0.75 to 15.39 ± 1.97 mg/g extract. The accumulation of quercetin aligns with the antioxidant trends in the TRI-NE extract, where the TPC increased by approximately 79% during fermentation (315.30 to 564.70 mg GAE/g), alongside parallel enhancements in DPPH, ABTS, and FRAP scavenging capacities. These findings indicate that the tri-culture microbial consortium induces a metabolic redistribution within the extract, specifically shifting the bioactive profile toward a concentrated abundance of high-potency reducing agents.

HFDPCs support neighboring fibroblasts during the anagen phase of the hair cycle through the secretion of key molecular ligands such as VEGF and Wnt signaling proteins [35]. Furthermore, this paracrine-mediated mechanism is supported by our gene expression analysis, which revealed a significant upregulation of *VEGF* and *CTNNB1* mRNA levels in HFDPCs following TRI-NE treatment (as discussed in Section 2.10). The ability of the TRI-NE-conditioned medium to outperform minoxidil in stimulating hTERT fibroblast proliferation in this study may be attributed not only to the increased quercetin content of the fermented extract but also to the synergistic effects of the enriched total phenolics. This observation is consistent with previous studies reporting that quercetin promotes cell proliferation in follicle-associated cellular models [36]. Overall, while HFDPC-derived paracrine signaling plays a central role in coordinating the activity of surrounding follicular cells during hair growth, our findings suggest that tri-culture fermentation significantly enhances this growth factor-mediated communication.

### 2.6. K_ATP_-Associated Cytoprotection of HFDPCs by Neem Extracts

**Figure 4:**
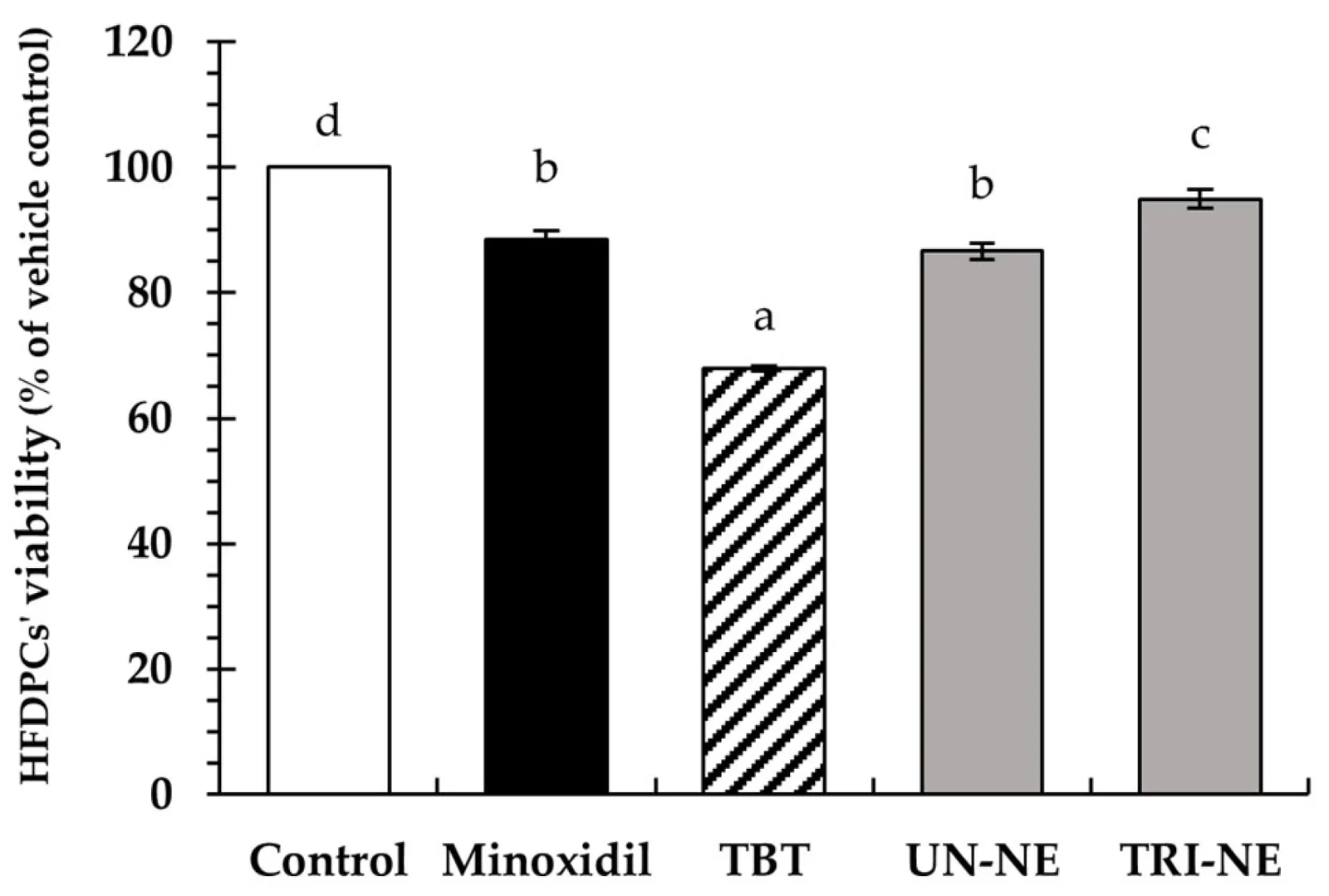
K_ATP_-associated cytoprotective effects of tri-culture fermented neem extract (TRI-NE) and unfermented neem extract (UN-NE) on HFDPC viability under pharmacological K_ATP_ channel blockade. Cells were treated with extracts or a standard treatment (minoxidil) at 0.0625 mg/mL in the presence of tolbutamide (TBT), relative to the negative control (TBT-treated cells). Values are expressed as mean ± SD (*n* = 3). One-way ANOVA followed by Tukey’s HSD post hoc test was applied; groups sharing no common letter (a–d) differ significantly (*p* < 0.05).

Minoxidil is widely recognized as a potassium channel opener. ATP-sensitive potassium (K_ATP_) channel modulation has been implicated in HFDPCs, which contributes to the regulation of cell survival, proliferation, and growth factor secretion [36]. To investigate the biological effects of extracts on a K_ATP_-dependent mechanism, TBT (tolbutamide) was employed as a pharmacological blocker of K_ATP_ channels and a mechanistic perturbation model. In this study, TBT exposure alone reduced HFDPC viability to 67.90 ± 0.40% of the control level, consistent with disruption of K_ATP_-dependent signaling associated with membrane potential regulation and cell survival (Figure 4) [37,38]. In contrast, TRI-NE extract restored HFDPC viability to 94.95 ± 1.46%, significantly exceeding the response observed with minoxidil (88.50 ± 1.41%) and UN-NE (86.57 ± 1.23%), respectively. All treated groups showed significant improvement compared with the TBT-only condition. These findings indicate that all the extracts, particularly TRI-NE, preserve HFDPC viability under pharmacological K_ATP_ channel blockade, suggesting a potential involvement of K_ATP_-associated cellular survival mechanisms.

The superior activity of TRI-NE extract cannot be attributed simply to increased polyphenol compound abundance following fermentation, as TRI-NE contained lower levels of various quantified polyphenols than UN-NE. The only major quantified compound increased after fermentation was quercetin, which has been reported to influence potassium-channel regulation and cellular redox processes that may affect K_ATP_ channel activity [39]. This selective enrichment of quercetin is consistent with the broader untargeted metabolomic profile, in which 67.47% of significantly altered metabolites were up-regulated following fermentation, indicating that fermentation selectively reshaped the metabolite composition rather than uniformly increasing the abundance of all metabolites and that a quercetin-enriched chemical profile may consequently contribute to the stronger functional activity observed in TRI-NE compared with the compositionally broader but less bioactive UN-NE. Collectively, these findings suggest that fermentation-driven functional enhancement may depend more on qualitative metabolite transformation than on overall polyphenol accumulation.

Furthermore, the markedly higher antioxidant capacities of TRI-NE extract compared with the UN-NE extract suggest that additional non-polyphenol reducing compounds generated during fermentation may contribute to cellular protection. These compounds may include organic acids and cell-wall degradation products associated with microbial metabolism during fermentation, providing complementary antioxidant support beyond the contribution expected from quercetin alone. In addition, these findings are consistent with our gene expression analysis, which revealed a significant upregulation of *VEGF* and *CTNNB1* mRNA levels in HFDPCs following TRI-NE treatment (as discussed in Section 2.10), providing molecular evidence that complements the K_ATP_-associated viability findings and further linking the observed functional protection to growth-promoting signaling pathways relevant to hair follicle biology. K_ATP_ channel activity in HFDPCs is associated with cellular states that favor continued proliferation and follicular growth signaling; the ability of TRI-NE to preserve HFDPC viability under K_ATP_ inhibition highlights a functional advantage conferred by tri-culture fermentation [38]. 

### 2.7. Anti-Inflammatory Properties of Neem Extracts in Cell-Based Models

**Figure 5:**
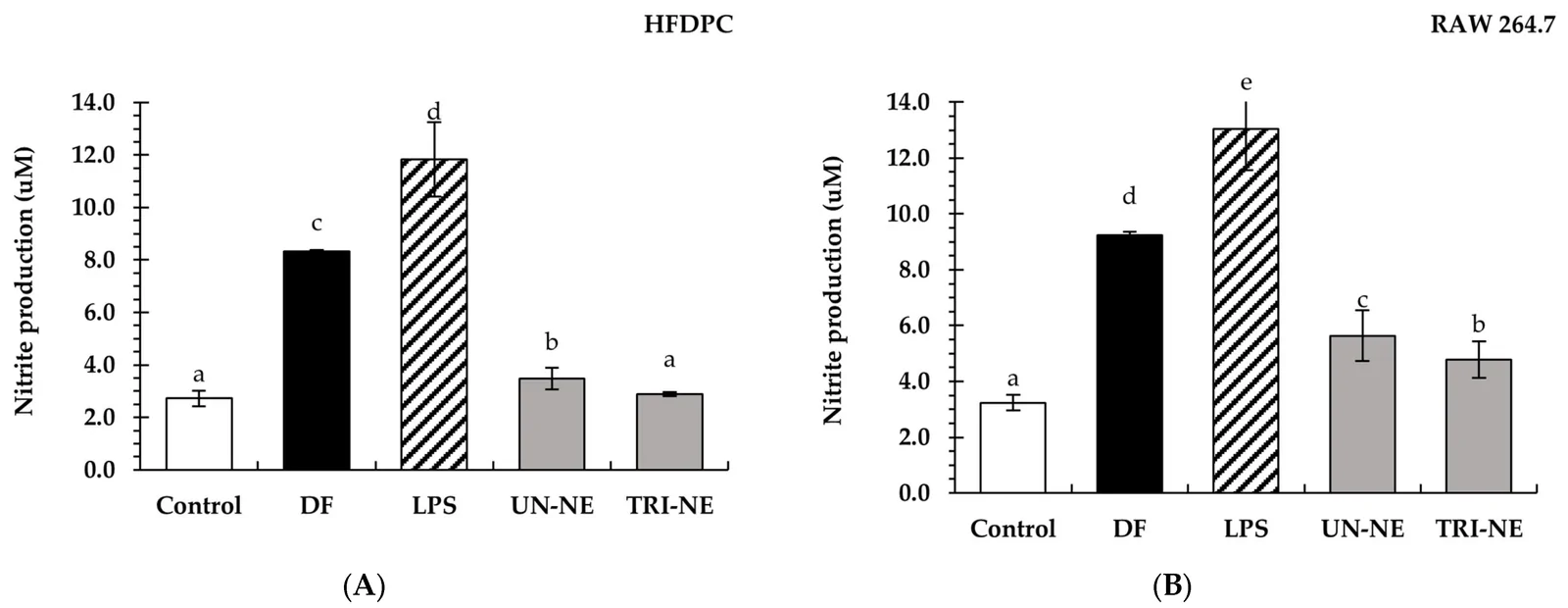
Anti-inflammatory effects of tri-culture fermented neem extract (TRI-NE) and unfermented neem extract (UN-NE) assessed by nitric oxide (NO) production in lipopolysaccharide (LPS)-stimulated RAW 264.7 macrophages (**A**) and HFDPCs (**B**). Cells were exposed to TRI-NE, UN-NE, or diclofenac sodium (DF) at 0.0625 mg/mL and evaluated against untreated cells and LPS-challenged cells without extract treatment. Data are presented as mean ± SD (*n* = 3). Statistical significance was determined using one-way ANOVA with Tukey’s HSD post hoc analysis; groups assigned different letters (a–e) indicate significant differences (*p* < 0.05).

Nitric oxide (NO) is a key inflammatory mediator. Excessive NO production in HFDPCs has been implicated in perifollicular inflammation and progressive hair follicle miniaturization in AGA [39]. In this study, LPS stimulation markedly elevated nitrite levels in both HFDPCs (11.83 ± 1.42 µM) and RAW 264.7 macrophages (13.04 ± 1.47 µM) relative to the untreated control, confirming successful induction of inflammatory responses in both cell models (Figure 5). Diclofenac sodium (standard anti-inflammatory drug) significantly reduced nitrite levels relative to the LPS-treated group, supporting the suitability of inflammatory models for evaluating anti-inflammatory effects. The parallel use of a hair follicle-derived cell type and a prototypical macrophage line thus provides complementary insights into the anti-inflammatory potential of neem extracts in both perifollicular and immune contexts.

Interestingly, neem extracts significantly suppressed nitrite levels in both cells compared with the LPS-treated group and diclofenac sodium (*p* < 0.05). Notably, TRI-NE exhibited the strongest suppression of nitrite production, with nitrite levels of 2.88 ± 0.08 µM in HFDPCs and 4.78 ± 0.65 µM in RAW 264.7 macrophages. This consistent response across both cell types suggests that TRI-NE exerts anti-inflammatory activity across different biological systems rather than in a cell-type-specific manner.

Furthermore, the superior NO-suppressing activity of TRI-NE may not be attributed solely to an overall increase in polyphenol content following fermentation. As shown in Section 2.3 (polyphenol quantification analysis), TRI-NE contained lower polyphenol levels compared with UN-NE. However, fermentation resulted in selective enrichment of specific bioactive constituents, particularly quercetin, which increased from 10.23 ± 0.75 mg/g extract in UN-NE to 15.39 ± 1.97 mg/g extract in TRI-NE. Quercetin has been reported to suppress *iNOS* expression and NO production through inhibition of NF-κB signaling and downregulation of pro-inflammatory transcription pathways [40], potentially contributing to the enhanced anti-inflammatory activity of TRI-NE despite its comparatively lower total polyphenol content. This finding is also consistent with the enhanced antioxidant profile and increased TPC observed following neem fermentation. TRI-NE exhibited improved antioxidant capacity compared with the unfermented extract, suggesting that microbial transformation may enrich bioactive metabolites with redox-regulatory potential. These enhanced antioxidant properties may contribute to the attenuation of oxidative stress-associated inflammatory signaling, thereby supporting the reduced inflammatory mediator production observed following TRI-NE treatment. In particular, enhanced FRAP activity may reflect the contribution of redox-active phenolic metabolites generated during fermentation, which could further support the cellular antioxidant environment associated with improved NO suppression by TRI-NE.

These findings align with the untargeted metabolomic analysis, which indicated that 67.47% of statistically significant metabolites increased in abundance after fermentation. These results indicate that microbial fermentation selectively reshapes the metabolite profile rather than uniformly enhancing all phenolic constituents. It is therefore plausible that additional fermentation-derived metabolites act in concert with quercetin to produce the enhanced anti-inflammatory activity of TRI-NE. Such compounds may act through complementary antioxidant or enzyme-modulatory mechanisms in addition to the contribution of polyphenols, beyond direct polyphenol accumulation consistent with previous reports linking fermentation-derived low-molecular-weight metabolites to enhanced NO-scavenging and iNOS-inhibitory activity [41].

These results demonstrate that neem extracts possess potent NO-suppressing activity significantly superior to the standard drug DF, with the tri-culture fermented extract (TRI-NE) exhibiting the strongest effect in both HFDPCs and RAW 264.7 cells. The functional enhancement observed after fermentation appears to arise from qualitative, rather than purely quantitative, transformation of the metabolite profile, supported by the parallel antioxidant enhancement observed across TPC, DPPH, ABTS, and FRAP assays. These findings reinforce the concept that microbial fermentation can generate a more bioactive extract through selective metabolite enrichment rather than simple polyphenol accumulation.

### 2.8. Suppression of Lipid Peroxidation in H_2_O_2_-Challenged HFDPCs by Neem Extracts

**Figure 6:**
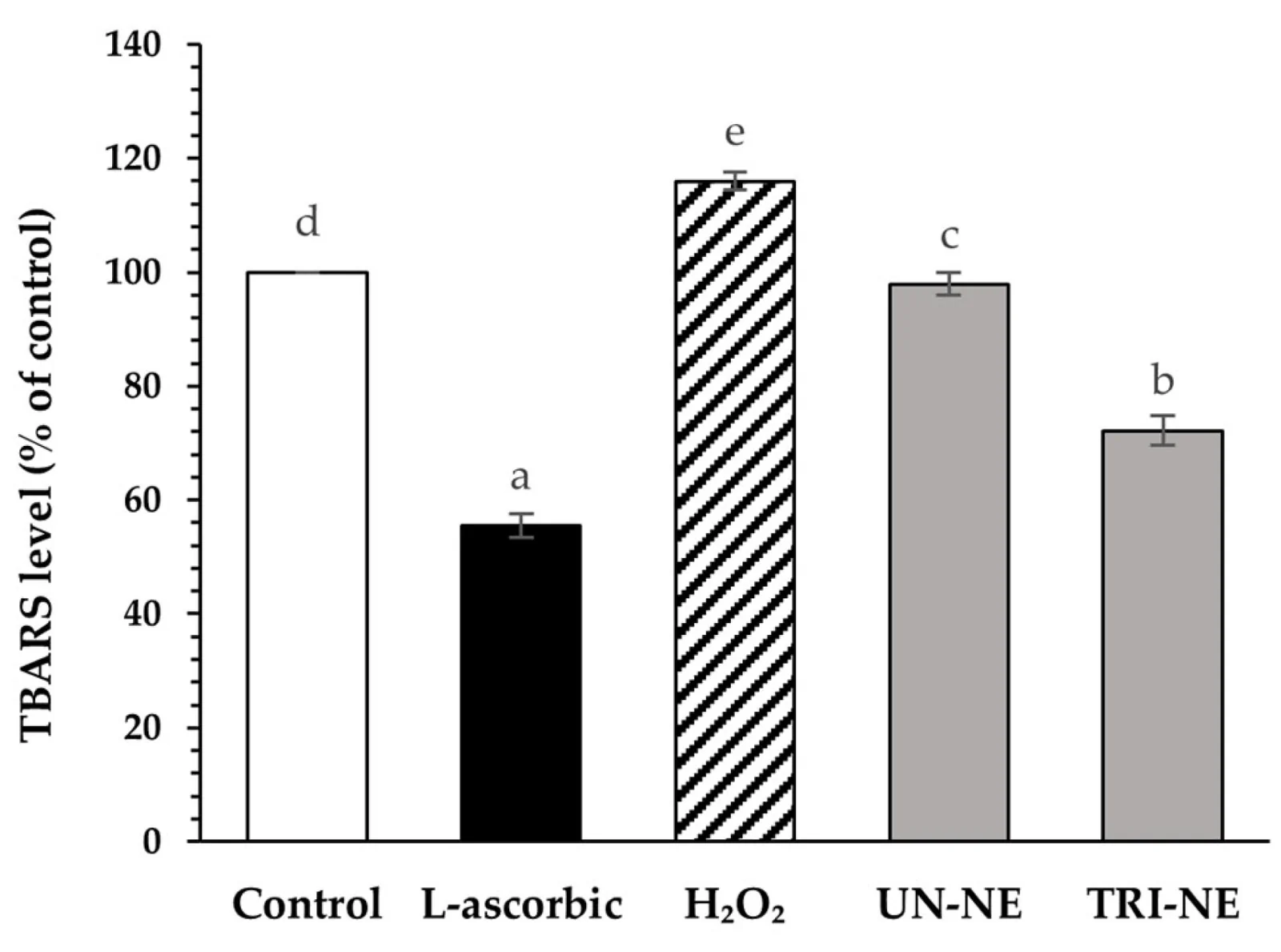
Reduction in oxidative lipid damage by tri-culture fermented neem extract (TRI-NE) and unfermented neem extract (UN-NE) in hydrogen peroxide (H_2_O_2_)-challenged HFDPCs, determined by thiobarbituric acid reactive substances (TBARS) levels. Cells were pre-treated with TRI-NE, UN-NE, or a reference antioxidant treatment (L-ascorbic acid) at 0.0625 mg/mL and compared with untreated controls under H_2_O_2_-induced oxidative stress conditions without extract treatment. Results are shown as mean ± SD (*n* = 3). Statistical differences were analyzed by one-way ANOVA followed by Tukey’s HSD post hoc analysis; different letters (a–e) denote significant differences among groups (*p* < 0.05).

Lipid peroxidation is a key target of oxidative stress, in which reactive oxygen species attack polyunsaturated fatty acids within cellular membranes, leading to the generation of malondialdehyde (MDA) as a stable end product that is widely used as a biomarker of oxidative membrane damage [42]. In this study, H_2_O_2_ exposure significantly increased TBARS levels relative to the untreated control, confirming the effective induction of oxidative injury in HFDPCs (Figure 6). In contrast, L-ascorbic acid (a reference antioxidant standard) significantly decreased TBARS levels to values comparable to the control, supporting the suitability of this oxidative stress model for evaluating antioxidant efficacy and providing a pharmacological benchmark for the performance of neem-derived formulations. Moreover, neem extracts significantly reduced TBARS levels relative to the H_2_O_2_-treated group. Particularly, TRI-NE produced a markedly stronger reduction, reaching 72.18 ± 2.65% of the control, approaching the efficacy of L-ascorbic acid while outperforming UN-NE. These results indicate that fermentation significantly elevated the lipid peroxidation-suppressing and membrane-protective capacities of the neem extract relative to the unfermented control and highlight TRI-NE as the most promising candidate for protecting hair follicle-derived cells from oxidative membrane damage.

Similar to the trends observed in NO suppression, the superior anti-lipid-peroxidative activity of TRI-NE cannot be explained by the overall polyphenol content alone. Instead, the selective enrichment of quercetin during fermentation may contribute through a distinct biophysical mechanism. Quercetin has been well documented to intercalate into lipid bilayers and directly scavenge peroxyl radicals, thereby interrupting the propagation phase of lipid peroxidation [43]. This protective mechanism is different from the previously discussed NF-κB/iNOS pathway but acts in a complementary manner. Collectively, these findings suggest that quercetin may contribute to both suppression of inflammatory signaling and protection against lipid peroxidation through distinct yet complementary mechanisms.

Furthermore, the TBARS-suppressing activity of TRI-NE aligns with the elevated antioxidant capacity of the fermented neem extract observed in our study. The fermentation increased radical-scavenging and reducing power relative to the unfermented extract. Since lipid peroxidation propagates through radical chain reactions, an extract with greater radical-quenching capacity should more effectively terminate this cascade before extensive MDA accumulates. The alignment between the cell-free antioxidant assays and the cell-based TBARS outcome strengthens the interpretation that fermentation-derived TRI-NE possesses membrane-protective antioxidant activity. These findings demonstrate that its robust in vitro radical-scavenging profile successfully translates to a cellular context. Therefore, the superior capability of TRI-NE to mitigate lipid peroxidation, compared to UN-NE, reinforces that the qualitative shift in the phytochemical profile—specifically the enrichment of key bioactive metabolites like quercetin—underlies the enhanced cytoprotective effect against H_2_O_2_-induced oxidative damage in HFDPCs.

### 2.9. Suppression of Intracellular Reactive Oxygen Species by Neem Extracts

**Figure 7:**
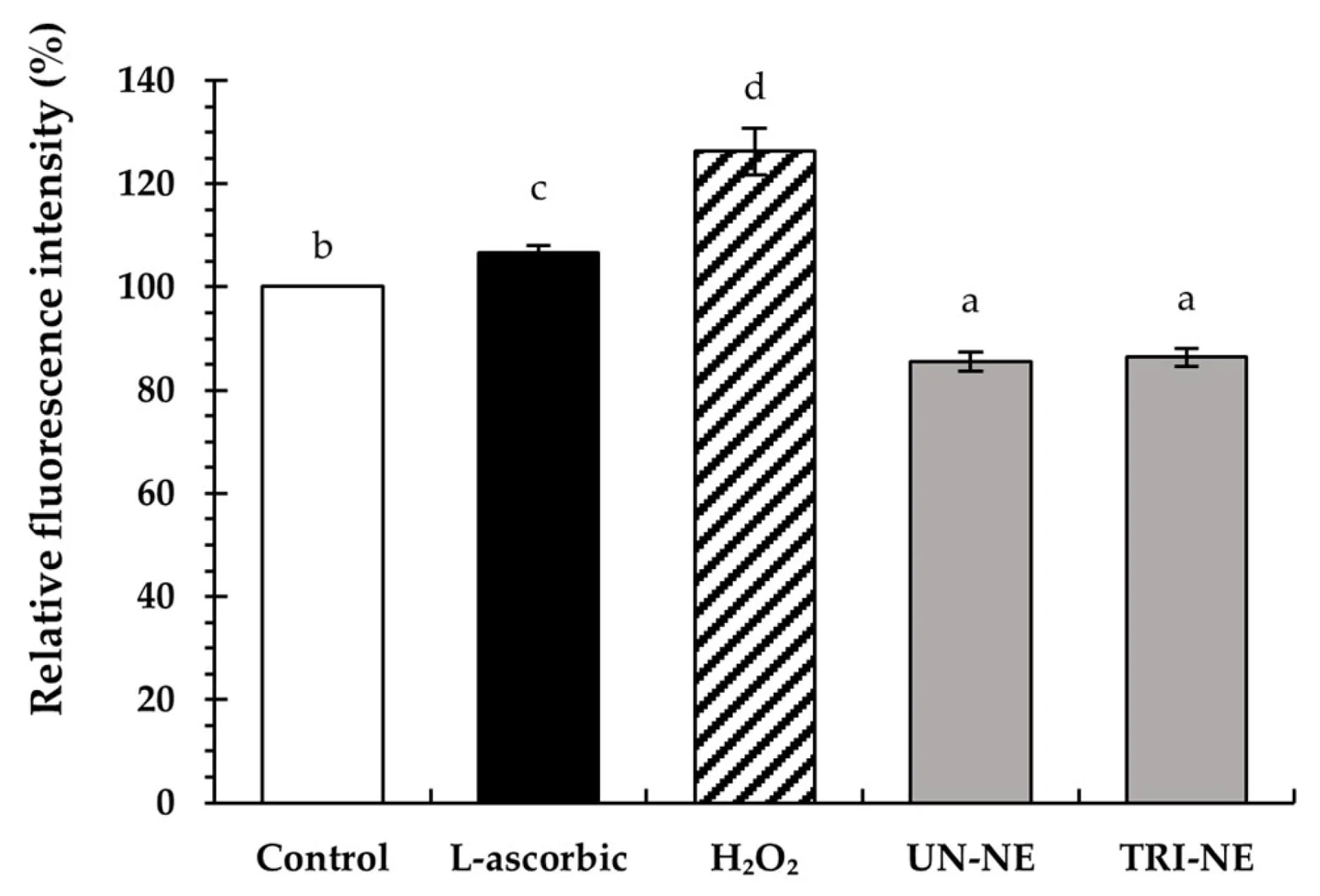
Protective effects of tri-culture fermented neem extract (TRI-NE) and unfermented neem extract (UN-NE) against intracellular reactive oxygen species (ROS) accumulation in hydrogen peroxide (H_2_O_2_)-stressed HFDPCs. Cells were pre-treated with TRI-NE, UN-NE, or L-ascorbic acid at 0.0625 mg/mL prior to H_2_O_2_ exposure. The results were compared with untreated cells and H_2_O_2_-exposed cells without extract treatment. Data are expressed as mean ± SD (*n* = 3). One-way ANOVA followed by Tukey’s HSD post hoc analysis was used for statistical evaluation; groups marked with different letters (a–d) represent significant differences (*p* < 0.05).

Intracellular reactive oxygen species (ROS) accumulation represents a cellular oxidative burden and serves as an early indicator of oxidative stress in HFDPCs. Excessive ROS accumulation has been associated with premature entry into the catagen phase and suppression of anagen-associated growth factor signaling, thereby contributing to hair follicle miniaturization and hair loss progression in AGA [42]. Conversely, maintaining ROS levels within a physiologically tolerable range is important for preserving HFDPC cellular functions and hair growth-associated signaling under oxidative stress conditions [42]. In this study, H_2_O_2_ significantly elevated intracellular ROS levels to 126.2 ± 4.46% of the untreated control, confirming successful induction of oxidative stress at the cellular level (Figure 7).

L-ascorbic acid (a reference antioxidant standard) significantly decreased ROS levels to values comparable to the control, supporting the suitability of this oxidative stress model for evaluating antioxidant efficacy and providing a pharmacological benchmark for the performance of neem-derived formulations. Neem extracts significantly reduced ROS levels relative to the H_2_O_2_-treated group. Interestingly, all neem extracts significantly reduced ROS levels below those of the untreated control. Based on these findings, the extracts may exhibit a potent antioxidant effect under high peroxide conditions or interact synergistically to inhibit baseline intracellular ROS generation without inducing cytotoxicity. Particularly, all neem extracts (TRI-NE and UN-NE) produced a markedly stronger reduction, reaching 85.5 ± 1.86% and 86.4 ± 1.77% of control (with no significant difference between the two extracts), respectively. This consistent reduction parallels the trends observed in the TBARS assay, indicating an effective attenuation of initial oxidative damage and downstream lipid peroxidation. Moreover, suppressing these oxidative markers below untreated control levels suggests that these treatments not only counteracted the exogenous H_2_O_2_ challenge but also down-regulated baseline metabolic ROS generation without affecting cell viability. Collectively, these findings demonstrate a comprehensive, multi-targeted antioxidant protection capable of preserving the redox homeostasis of HFDPCs.

Interestingly, despite TRI-NE containing higher quercetin and showing stronger activity in NO and TBARS assays, both TRI-NE and UN-NE exhibited similar ROS suppression. This suggests that intracellular ROS regulation in HFDPCs is not driven by a single dominant compound enriched through fermentation. Instead, it likely reflects the combined effects of a diverse metabolite profile. Untargeted metabolomics revealed that fermentation altered thousands of metabolites, with 67.47% significantly upregulated, supporting the idea that multiple constituents—both pre-existing and fermentation-derived—collectively contribute to ROS-scavenging capacity.

From a hair biology perspective, the ability of neem extracts to reduce ROS below baseline levels indicates a potential to alleviate oxidative burden beyond normalization. This may help maintain a redox environment favorable for anagen phase maintenance and paracrine growth factor signaling. Together with the TBARS and NO findings, these results support the role of neem extracts—regardless of fermentation status—as effective protectants against oxidative stress in HFDPCs, while fermentation appears to provide additional advantages in more specific pathways such as lipid peroxidation and inflammatory signaling.

### 2.10. Modulation of Hair Regeneration Gene Expression by Neem Extracts

Hair regeneration is a complex biological process consisting of four phases: growth (anagen), regression (catagen), rest (telogen), and shedding (exogen). The progression of the hair cycle is regulated by multiple signaling pathways that collectively control hair follicle activity [29,44]. In the present study, we evaluated the major signaling pathways involved in hair regeneration following treatment with neem extracts, including pathways associated with hair loss progression, such as the androgen and transforming growth factor-β (TGF-β) pathways, as well as hair growth-promoting pathways, including Wnt/β-catenin, Sonic Hedgehog, and angiogenesis pathways.

**Figure 8:**
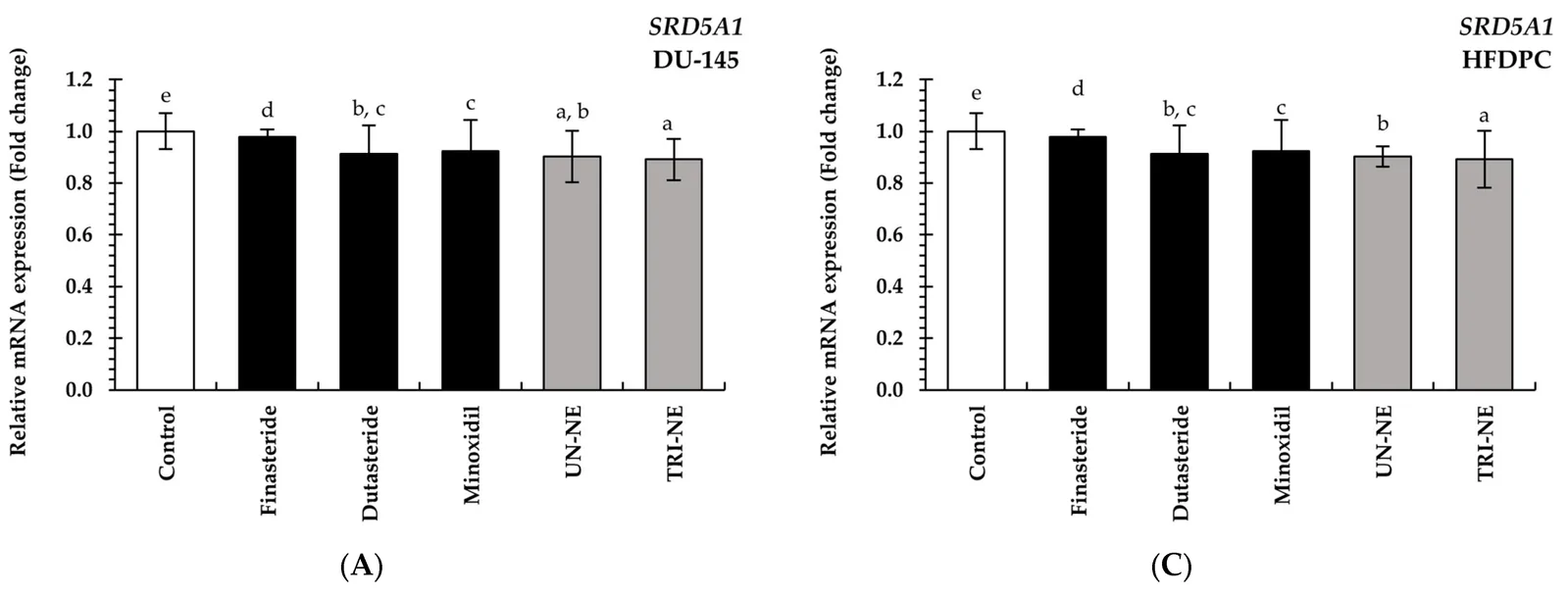

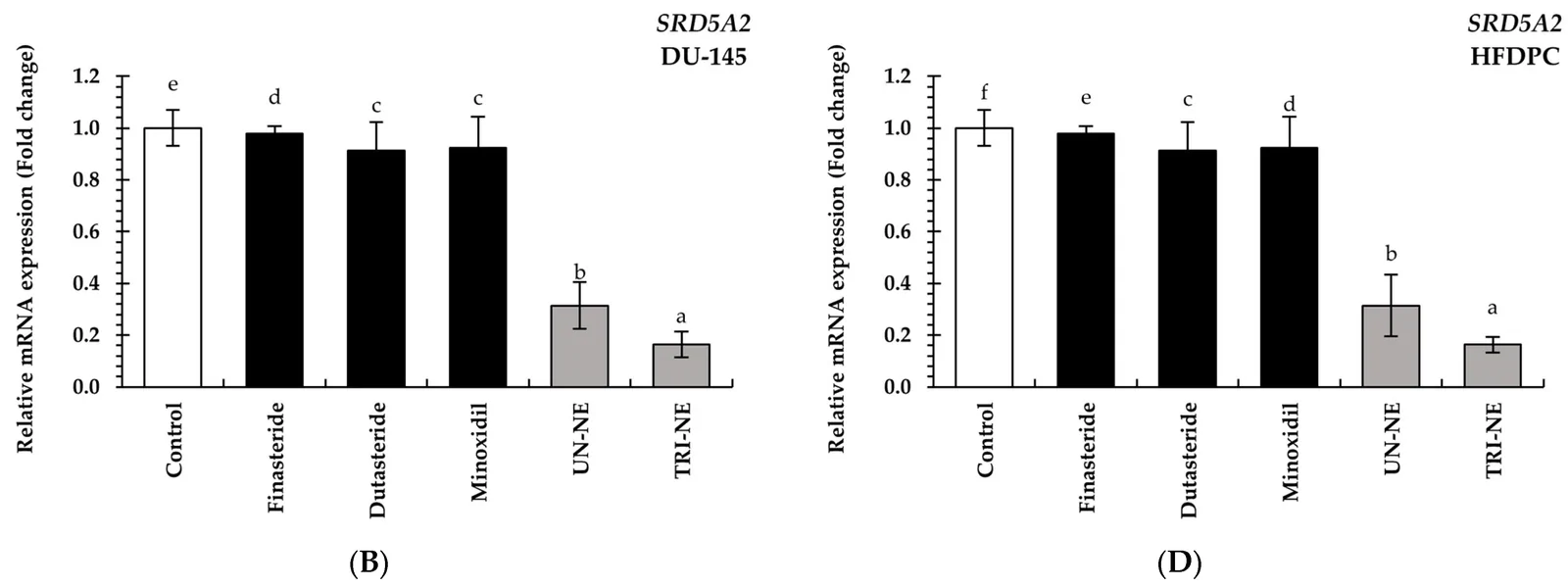
Expression profiles of androgen-related genes following treatment with tri-culture fermented neem extract (TRI-NE) and unfermented neem extract (UN-NE) in DU-145 cells and HFDPCs. Relative mRNA levels of *SRD5A1* and *SRD5A2* were quantified in DU-145 cells (**A**,**B**) and HFDPCs (**C**,**D**). Cells were treated with TRI-NE, UN-NE, or pharmacological reference compounds (dutasteride, finasteride, and minoxidil) at 0.0625 mg/mL. Gene expression was normalized to *GAPDH*. Data are presented as mean ± SD (*n* = 3). Statistical analysis was performed using one-way ANOVA with Tukey’s HSD post hoc analysis; different letters (a–f) indicate significant differences between groups (*p* < 0.05).

DHT is an androgenic steroid hormone generated through the action of SRD5A enzymes, which convert testosterone into DHT. Excessive DHT accumulation in androgen-sensitive regions of the scalp shortens the anagen phase of the hair cycle, resulting in premature follicular miniaturization and eventual hair loss [44,45]. Fermentation of neem extract with a tri-culture of *S. cerevisiae*, *L. plantarum*, and *A. niger* increased quercetin content in TRI-NE compared with UN-NE. Previous studies have reported that quercetin can inhibit SRD5A enzymatic activity [28]. Based on this rationale, the effects of UN-NE and TRI-NE on the expression of androgen pathway-related genes (*SRD5A1* and *SRD5A2*) were first evaluated in DU-145 cells and HFDPCs in comparison with the standard drugs for hair loss treatment, including finasteride, dutasteride, and minoxidil (Figure 8). All neem extracts significantly suppressed *SRD5A1* and *SRD5A2* expression compared with the untreated control in both cell types (*p* < 0.05) (Figure 8). TRI-NE demonstrated the strongest suppression of *SRD5A1* and *SRD5A2* expression among all tested groups, with inhibitory effects approximately 1.20-fold, 1.65-fold, and 1.75-fold greater than those observed with dutasteride, minoxidil, and finasteride, respectively. Moreover, UN-NE also significantly suppressed *SRD5A1* and *SRD5A2* expression compared with the untreated control, although its effects differed from those observed with the standard drugs. Notably, TRI-NE exerted a stronger suppressive effect on *SRD5A* expression in HFDPCs than in DU-145 cells. HFDPCs are a hair follicle-relevant cell model, whereas DU-145 cells are commonly used for evaluating 5α-reductase-related activity. Nevertheless, both cell models showed a similar response pattern across all tested samples, supporting the consistent inhibitory activity of TRI-NE against *SRD5A* expression.

**Figure 9:**
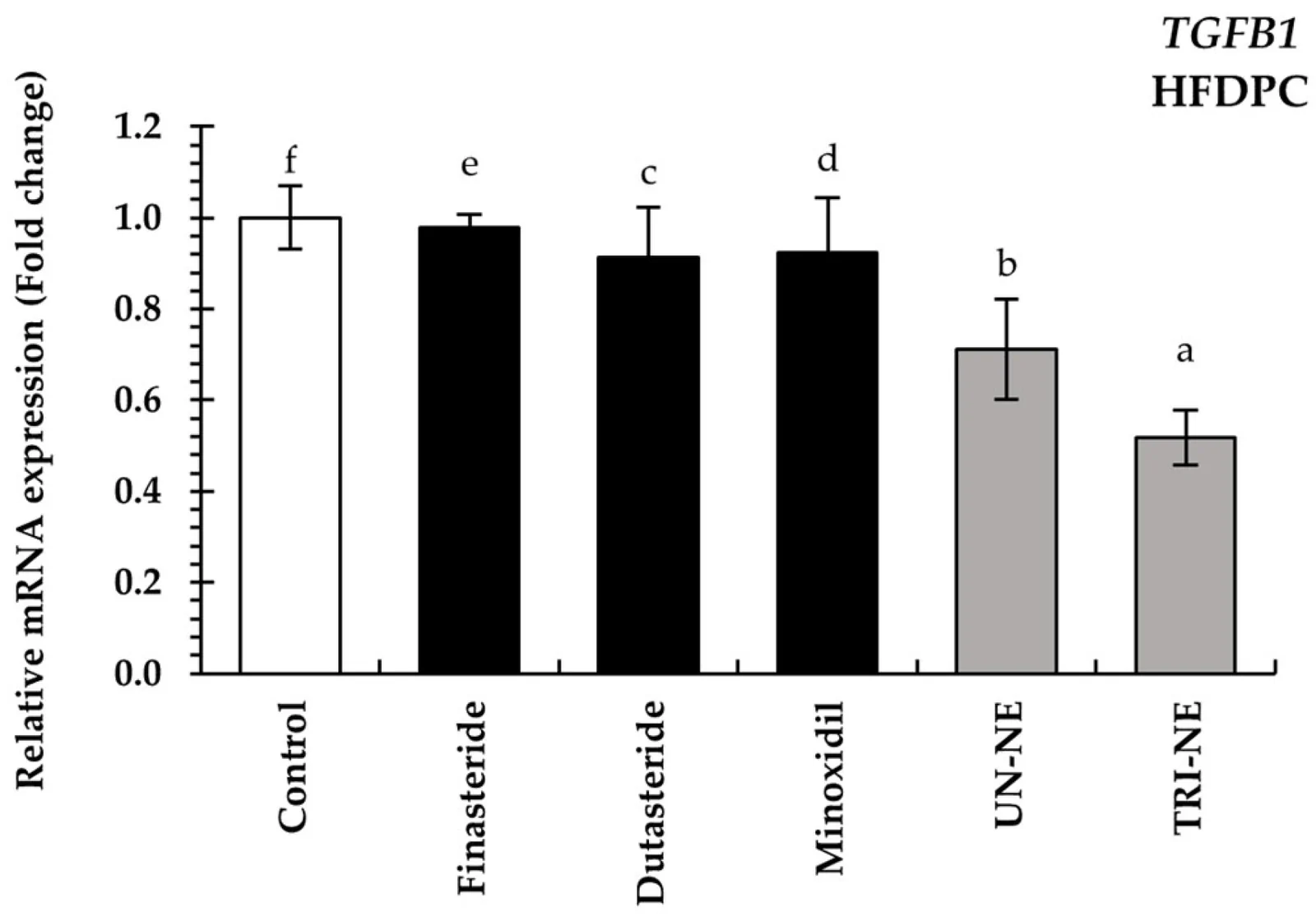
Transcriptional response of the TGF-β signaling marker *TGFB1* in HFDPCs following treatment with tri-culture fermented neem extract (TRI-NE), unfermented neem extract (UN-NE), or pharmacological reference treatments (dutasteride, finasteride, and minoxidil) at 0.0625 mg/mL. Relative *TGFB1* mRNA expression was normalized against *GAPDH*. Values are expressed as mean ± SD (*n* = 3). Statistical significance was assessed using one-way ANOVA followed by Tukey’s HSD post hoc analysis; groups with different letters (a–f) represent significant differences (*p* < 0.05).

DHT also stimulates the expression of *TGFB1* in HFDPCs, which subsequently induces apoptotic signaling in follicular epithelial cells and accelerates the transition from anagen to catagen, thereby contributing to hair loss [45]. In our study, neem extracts suppressed the upstream SRD5A enzymes responsible for DHT generation, and the downstream effects on *TGFB1* expression were subsequently examined. All neem extracts significantly decreased *TGFB1* expression compared with the untreated control and standard hair loss treatments (dutasteride, finasteride, and minoxidil) (*p* < 0.05) (Figure 9), with TRI-NE again showing the greatest suppression (0.52 ± 0.06-fold change relative to the untreated control), while the standard treatments also reduced *TGFB1* expression, including minoxidil (0.82 ± 0.10-fold change), finasteride (0.95 ± 0.08-fold change), and dutasteride (0.96 ± 0.10-fold change). The combined suppression of androgen pathway-related genes (*SRD5A1* and *SRD5A2*) and the downstream mediator gene *TGFB1* suggests that the anti-androgenic activity of TRI-NE is not restricted to a single component of the pathway but extends across both DHT biosynthesis and downstream signaling events associated with hair follicle miniaturization.

HFDPCs are central to hair follicle formation and mediate essential interactions between mesenchymal, epithelial, and fibroblast cells within the hair follicle microenvironment [2,44]. The Wnt/β-catenin (*CTNNB1*) signaling pathway is a key driver of HFDPC proliferation. Normally, Wnt ligand binding to Frizzled and LRP co-receptors stabilizes β-catenin against proteasomal degradation. Subsequently, stabilized β-catenin translocates into the nucleus, followed by the activation of genes supporting proliferation and migration relevant to hair growth. The Wnt/β-catenin pathway acts upstream of the Sonic Hedgehog pathway, which mediates signal transduction between mesenchymal and epithelial cells. The Sonic Hedgehog pathway also plays an important role in the transition from telogen to anagen. In the canonical Hedgehog cascade, SHH ligand binding to the Patched (PTCH) receptor relieves the repression of Smoothened (SMO), which subsequently activates GLI family transcription factors. Activated GLI factors then translocate to the nucleus to drive the transcription of genes supporting follicular development and stem cell maintenance [2,44]. In addition, VEGF plays a vital role in promoting angiogenesis during the anagen phase, enhancing oxygen and nutrient delivery to the follicle and supporting increases in follicle diameter [46].

**Figure 10:**
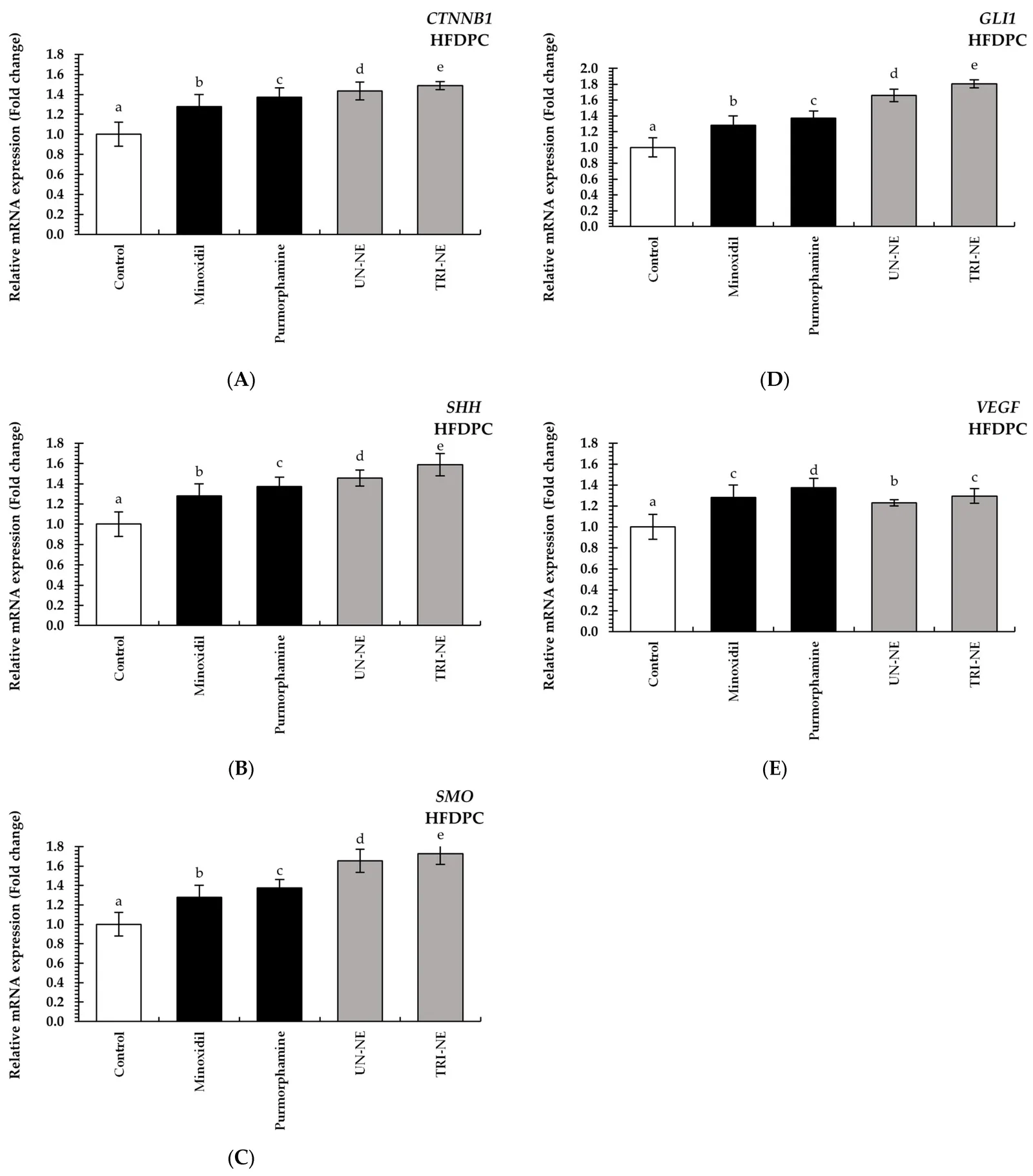
Regulation of hair growth-associated transcriptional markers in HFDPCs treated with tri-culture fermented neem extract (TRI-NE), unfermented neem extract (UN-NE), and standard compounds (minoxidil and purmorphamine) at 0.0625 mg/mL. Relative mRNA expression was evaluated for genes involved in Wnt/β-catenin signaling (**A**) *CTNNB1*, Sonic Hedgehog signaling (**B**) *SHH*, (**C**) *SMO*, and (**D**) *GLI1*, and angiogenic regulation (**E**) *VEGF*. Expression levels were normalized to *GAPDH* and compared among treatment groups. Data are reported as mean ± SD (*n* = 3). One-way ANOVA with Tukey’s HSD post hoc analysis was used; groups represented by different letters (a–e) are statistically distinct (*p* < 0.05).

In this study, both neem extracts significantly upregulated *CTNNB1* expression compared to the untreated control (Figure 10A). Furthermore, TRI-NE (1.49 ± 0.04) significantly upregulated *CTNNB1* expression compared to standard treatments of minoxidil and purmorphamine (*p* < 0.05). A similar pattern was observed across the Hedgehog pathway components, with TRI-NE achieving the highest fold-changes for *SHH*, *SMO*, and *GLI1* (Figure 10B–D). TRI-NE achieved the highest upregulated fold-changes for *SHH* (1.59 ± 0.11), *SMO* (1.73 ± 0.11), and *GLI1* (1.81 ± 0.05), significantly greater than UN-NE in all Hedgehog pathway genes. Quercetin has previously been reported to promote cytoplasmic accumulation and nuclear translocation of β-catenin, thereby enhancing *CTNNB1* transcription, and separately to stimulate Sonic Hedgehog pathway activity in dermal papilla-derived cells [36]. In our study, TRI-NE contained a higher quercetin content than UN-NE despite lower levels of several other quantified polyphenols. This discrepancy highlights the broader impact of the tri-culture fermentation process, which transformed the global chemical landscape. Untargeted metabolomics confirmed that fermentation altered thousands of metabolites, with 67.47% of these significantly upregulated. This metabolic transformation indicates a general trend toward metabolite accumulation and the generation of new bioactives following microbial processing.

Furthermore, both extracts also significantly upregulated *VEGF* expression compared to the untreated control (*p* < 0.05) (Figure 10E), with TRI-NE showing the highest upregulated *VEGF* fold-change (1.29 ± 0.07), which was comparable to minoxidil (1.28 ± 0.12) with no significant difference, a well-established VEGF stimulator known to support angiogenesis during active hair regeneration [46]. These findings may be attributed to the combined effects of quercetin and other fermentation-derived metabolites that contribute to angiogenic signaling activation, consistent with the enhanced antioxidant capacity of TRI-NE described in the preceding sections.

Collectively, these findings demonstrate that TRI-NE showed stronger modulation of androgen, Wnt/β-catenin, Sonic Hedgehog, and angiogenesis-related gene expression than UN-NE and the standard hair loss treatments evaluated in this study. Although TRI-NE contained lower levels of several individual polyphenols, its superior biological activity suggests that tri-culture fermentation altered the neem chemical composition, resulting in a metabolite profile enriched in quercetin and other bioactive compounds that may contribute to the activation of multiple complementary signaling pathways relevant to hair follicle growth and regeneration.

Nevertheless, the present comparisons demonstrate enhanced phytochemical and biological properties of TRI-NE relative to UN-NE but do not establish that the addition of *A. niger* provides an incremental benefit over the previously employed *S. cerevisiae*–*L. plantarum* dual-culture system. A corresponding dual-culture neem treatment was not included in the present experimental design; therefore, the individual contribution of *A. niger* and potential synergistic interactions among the three microorganisms cannot be resolved. Future studies incorporating mono-, dual-, and tri-culture treatments under otherwise identical fermentation conditions will be required to distinguish strain-specific contributions and determine whether increasing consortium complexity provides additional functional benefits.

## 3. Materials and Methods

### 3.1. Chemicals and Reagents

All reagents used throughout the experiments were of analytical grade. The specific chemical compounds, cell culture media, cell lines, and software applications utilized in this study, along with their respective manufacturers and origins, are summarized in Supplementary Table S2.

### 3.2. Extract Preparation

#### 3.2.1. Neem: Source and Powder Preparation

Neem leaves were obtained from Mueang Mai Supplier, a local market vendor in Mueang District, Chiang Mai Province, Thailand, in October 2025. A voucher specimen (PNPRDU68002) was collected and authenticated by N. Panti and deposited at the Pharmaceutical and Natural Products Research and Development Unit (PNPRDU), Faculty of Pharmacy, Chiang Mai University, Thailand. Fresh material was washed thoroughly under running water to remove surface debris, drained, air-dried under sunlight to reduce the high initial moisture content of fresh plant material and minimize the risk of microbial spoilage prior to complete drying, and subsequently dried in a hot-air oven at 70 °C for 24 h, a temperature previously reported to be suitable for neem leaf processing and associated with high retention of bioactive compounds in this species [47,48,49,50]. The dried material was ground using a laboratory mill and passed through a 425 µm stainless steel mesh sieve to obtain a uniform fine powder, which was stored in sealed containers at room temperature, protected from light and moisture, until use.

#### 3.2.2. Microbial Strains: Source and Inoculum Preparation

The three microbial strains used in this study were purchased from the Thailand Institute of Scientific and Technological Research (TISTR): *S. cerevisiae* TISTR 5169, *L. plantarum* TISTR 2072, and *A. niger* TISTR 3056. All strains were maintained and subcultured according to TISTR standard protocols prior to experimental use.

Each microbial strain was individually cultured in its optimal liquid growth medium as follows. *S. cerevisiae* TISTR 5169 was cultured in Yeast Extract Peptone Dextrose (YPD) broth (10 g/L yeast extract, 20 g/L peptone, 20 g/L dextrose) and incubated at 30 °C for 24–48 h under aerobic conditions. *L. plantarum* TISTR 2072 was cultured in De Man, Rogosa, and Sharpe (MRS) broth (Himedia, India) and incubated at 37 °C for 24–48 h under anaerobic conditions. *A. niger* TISTR 3056 was cultured in Potato Dextrose Broth (PDB) (Himedia, India) and incubated at 30 °C for 2–5 days under aerobic conditions. Subsequently, microbial cells were harvested by centrifugation at 5000× *g* for 10 min at 4 °C and the resulting cell pellets were washed twice with sterile 0.85% (*w*/*v*) NaCl solution to remove residual growth medium. The washed cells were then subjected to freeze-drying to produce a dry powdered form of each microbial strain. The freeze-dried powders were stored at −20 °C until use.

Prior to inoculation, the freeze-dried powder of each strain was reconstituted individually in sterile distilled water to a concentration of 0.1% (*w*/*v*). Cell density of each reconstituted suspension was adjusted to equivalent concentrations. The three microbial suspensions were then combined in equal proportions (1:1:1, *v/v/v*) to yield the mixed-culture inoculum used for fermentation.

#### 3.2.3. Fermentation of Neem

Fermentation was conducted as a completely randomized design (CRD) with two treatment groups (an unfermented control, UN-NE, and a tri-culture-fermented, TRI-NE) and three biological replicates per treatment and sampling time. Independent fermentation jars were prepared for each treatment and sampling time to avoid repeated disturbance of the fermentation system. Fifty grams of neem powder were placed in a sterile glass fermentation jar, and a minimal volume of sterile distilled water was added and mixed thoroughly to form a homogeneous slurry. For the fermented treatment, the tri-culture inoculum (mixed-culture consortium of *S. cerevisiae*, *L. plantarum*, and *A. niger* at a 1:1:1 ratio) was prepared in a mineral salt solution (1% (*w*/*v*) glucose, 10% (*w*/*v*) KH_2_PO_4_, 0.25% (*w*/*v*) MgSO_4_, adjusted to pH 6.0) and added at a final inoculation rate of 1% (*v*/*w*); the control treatment received an equivalent volume of sterile mineral salt solution without microbial inoculum. Jars were sealed with gas-permeable caps and incubated at 30 °C for 7 days. After fermentation, all material was dried in a hot-air oven at 70 °C to constant weight, then milled and sieved through a 425 µm mesh to yield a fine powder, which was stored at −20 °C until extraction.

#### 3.2.4. Extraction

The dried plant powder was extracted using ultrasonic-assisted extraction (UAE). Each powder sample was mixed with 95% (*v*/*v*) ethanol at a ratio of 1:5 (*w*/*v*) and homogenized thoroughly. Extraction was performed using an ultrasonic bath (40 kHz) for 20 min under continuous cooling to minimize thermal degradation of heat-sensitive constituents. The extraction procedure was repeated twice to maximize the recovery of extractable compounds. The extracts were subsequently filtered through Whatman filter paper to remove particulate matter.

The combined filtrates were concentrated using a rotary evaporator (Rotavapor^®^ R-300, Büchi, Switzerland) under reduced pressure (200 mbar) at 30 °C and 120 rpm. Following solvent evaporation, each concentrated extract was adjusted to a final volume of 15 mL to provide a consistent stock volume for subsequent handling and analysis.

The dry extract content of each concentrated stock was estimated using a volumetric–gravimetric dry-residue method. Briefly, a 1.00 mL aliquot of each extract was transferred into a pre-weighed container and dried at 60 °C (*n* = 3). After drying and cooling, the container was reweighed, and the dry residue mass was determined by subtracting the weight of the empty container. The dry extract concentration was calculated as the mass of dry residue obtained per milliliter of extract. The total dry extract mass was subsequently estimated from the gravimetrically determined dry extract concentration and the total volume of the concentrated extract. Extraction yield was calculated relative to the initial dry weight of plant material used for extraction as follows.

$$\mathrm{Extraction}\;\mathrm{yield}\;\left(\%\right)=\frac{\mathrm{Estimated}\;\mathrm{total}\;\mathrm{dry}\;\mathrm{extract}\;\mathrm{mass}\;(g)}{\mathrm{Initial}\;\mathrm{dry}\;\mathrm{sample}\;\mathrm{mass}\;(g)}\;\times \;100$$

Only the defined aliquot used for gravimetric determination was subjected to drying, whereas the remaining concentrated extract was retained in liquid form, protected from light, and stored at 4 °C until subsequent analyses.

### 3.3. Phytochemical Analysis

#### 3.3.1. Determination of Total Phenolic Content (TPC)

Total phenolic content (TPC) of each sample was determined using the Folin–Ciocalteu colorimetric method according to the method described by [51] with slight modifications. Briefly, each sample solution was mixed with Folin–Ciocalteu reagent and allowed to react for 3 min at room temperature. Subsequently, 2% (*w*/*v*) Na_2_CO_3_ solution was added to the mixture and incubated at room temperature for 60 min. Absorbance was measured at 750 nm using a spectrophotometer (Thermofisher, Waltham, MA, USA). All assays were performed in triplicate. A standard calibration curve was constructed using gallic acid at a series of known concentrations, and TPC was expressed as milligrams of gallic acid equivalents per gram of extract (mg GAE/g).

#### 3.3.2. Antioxidant Activities

- ABTS Radical Scavenging Assay

Antioxidant activity by ABTS^+^• scavenging was assessed according to the previously described method [52] with slight modifications. A stock ABTS^+^• solution was prepared by mixing 7.4 mM ABTS solution with 2.45 mM K_2_S_2_O_8_ solution in a 1:1 (*v*/*v*) ratio. The mixture was stored in the dark at room temperature for 15–18 h to allow complete radical cation formation. Prior to use, the stock ABTS^+^• solution was diluted in methanol, and its absorbance was adjusted to 0.7 ± 0.2 at 750 nm to obtain the working ABTS^+^• reagent. Antioxidant activity of each extract was measured by mixing 100 μL of sample with 900 μL of ABTS^+^• working reagent, incubating in the dark at room temperature for 6 min, and measuring absorbance at 750 nm. Gallic acid (≥98.0% purity; Sigma-Aldrich, St. Louis, MO, USA) was used as the reference standard to construct the calibration curve. Accordingly, ABTS radical-scavenging capacity was calculated from the gallic acid calibration curve and expressed as milligrams of gallic acid equivalents per gram of extract (mg GAE/g). The use of gallic acid as the calibration standard represented a modification of the originally described ABTS method.

- DPPH Radical Scavenging Assay

Antioxidant activity by DPPH radical scavenging was evaluated according to the previously described method [53] with slight modifications. A DPPH working solution was prepared at a concentration of 0.2 mM in methanol. An aliquot of 100 μL of each sample solution was mixed with 900 μL of DPPH solution. The mixture solution was incubated in the dark at room temperature for 30 min, after which absorbance was measured at 517 nm using a spectrophotometer (Thermofisher, USA). All assays were performed in triplicate. Gallic acid was used as the reference standard to construct a calibration curve. DPPH radical-scavenging capacity was calculated from the gallic acid calibration curve and expressed as milligrams of gallic acid equivalents per gram of extract (mg GAE/g). The use of gallic acid as the calibration standard represented a modification of the originally described method.

- Ferric Reducing Antioxidant Power (FRAP) Assay

Ferric reducing antioxidant power (FRAP) was determined according to the method described by [54] with slight modifications. The FRAP reagent was freshly prepared by mixing 10 mM TPTZ (2,4,6-tripyridyl-s-triazine) dissolved in 40 mM HCl, 20 mM FeCl_3_·6H_2_O in distilled water, and acetate buffer (pH 3.6, prepared from sodium acetate and acetic acid) in a volumetric ratio of 1:1:10, respectively. For the assay, 100 μL of each sample solution was mixed with 900 μL of freshly prepared FRAP reagent and incubated at room temperature for 5 min. Absorbance was measured at 595 nm using a spectrophotometer (Thermofisher, USA). All assays were performed in triplicate. FRAP values were calculated from an Fe (II) calibration curve and expressed as μmol Fe^2+^ equivalents per gram of extract (μmol Fe^2+^/g extract).

### 3.4. Untargeted Metabolomic Profiling

#### 3.4.1. Metabolite Extraction and Sample Preparation

Metabolite extraction was conducted according to an established plant metabolomics procedure with slight modifications [55,56]. Samples were initially centrifuged at 14,000× *g* for 20 min at 16 °C to separate insoluble debris. The resulting supernatants were subsequently filtered through Amicon^®^ Ultra 3 kDa molecular-weight cut-off ultrafiltration membranes (UFC9003, Millipore, Burlington, MA, USA) to eliminate proteins and other high-molecular-weight constituents while retaining low-molecular-weight metabolites. The filtrates were purified using C18 solid-phase extraction (SPE) cartridges (Supelclean™, Sigma-Aldrich, Bellefonte, PA, USA) operated under vacuum. Prior to sample loading, cartridges were activated with 30 mL acetonitrile and equilibrated using 60 mL deionized water. Following sample application, cartridges were rinsed with water to remove residual impurities before metabolites were recovered with 99% acetonitrile. The eluates were evaporated to dryness under reduced pressure using a rotary evaporator. Dried residues were dissolved in 1 mL methanol and subsequently diluted with deionized water to obtain a final volume of 10 mL prior to LC–MS analysis.

#### 3.4.2. UHPLC–MS/MS Analysis

Untargeted metabolomic analysis was performed using an UltiMate 3000 ultra-high-performance liquid chromatography (UHPLC) system (Thermo Fisher Scientific, USA) coupled to a Q Exactive-X Orbitrap mass spectrometer (Thermo Fisher Scientific). Metabolites were separated on a Hypersil GOLD™ C18 analytical column (2.1 × 100 mm, 1.9 μm) maintained at 60 °C. Samples (3 μL) were introduced at a flow rate of 0.35 mL/min, while the autosampler temperature was maintained at 8 °C throughout the analytical sequence. Data acquisition was carried out under both positive and negative electrospray ionization (ESI) conditions. For positive ionization, mobile phase A consisted of methanol/water (10:90, *v*/*v*) containing 0.1% formic acid and 10 mM ammonium formate, whereas mobile phase B comprised acetonitrile/water (90:10, *v*/*v*) with identical additives. Under negative ionization, solvent A contained methanol/water (10:90, *v*/*v*) supplemented with 0.1% acetic acid, while solvent B consisted of acetonitrile/water (90:10, *v*/*v*) containing 0.1% acetic acid. Chromatographic separation employed a gradient beginning at 5% B (95% A) for 2 min, followed by a linear increase to 55% B over 6 min. The column was subsequently washed with 99% B for 5 min before returning to the initial mobile-phase composition for column re-equilibration, resulting in a total run time of 20 min. Methanol blanks were injected between consecutive biological samples to minimize carryover.

The mass spectrometer operated with spray voltages of 3.5 kV in positive mode and 3.2 kV in negative mode. Sheath and auxiliary gas flow rates were set to 50 and 10 arbitrary units, respectively, while the capillary temperature was maintained at 350 °C. Full-scan MS data were collected over an *m*/*z* range of 85–850 at a resolving power of 180,000 (*m*/*z* 200). Data-dependent MS/MS acquisition selected the twenty most abundant precursor ions from each survey scan using a 1.4 Da isolation window, followed by higher-energy collisional dissociation (HCD). Fragment spectra were acquired at a resolution of 30,000 with dynamic exclusion enabled to reduce repeated fragmentation of identical precursor ions. Instrument control and data collection were performed using Xcalibur version 3.1 (Thermo Fisher Scientific).

#### 3.4.3. Data Processing and Metabolite Annotation

Raw UHPLC–MS/MS datasets were processed using Compound Discoverer version 3.1 (Thermo Fisher Scientific) following a standard untargeted metabolomics workflow. Peak detection and chromatographic alignment were conducted using a retention time tolerance of 0.4 min and a mass tolerance of 5 ppm. Only features exceeding a signal-to-noise ratio of 1.5 were retained, while an intensity variation threshold of 30% was applied during feature alignment. To improve analytical reproducibility, pooled quality-control (QC) samples were used for signal normalization through cubic spline regression. Features exhibiting intensities below 5 × 10^5^ arbitrary units were removed prior to downstream analysis. Putative molecular formulas were assigned based on accurate mass measurements and isotopic distribution patterns within a 5 ppm mass error threshold. Compound annotation was subsequently performed through spectral matching against the mzCloud database (accessed on 18 March 2026). Additional candidate identities were evaluated using ChemSpider, incorporating records from databases including ChEBI and ChemBank [57]. Natural-product annotations were further verified against The Natural Products Atlas version 3.0 (accessed on 18 March 2026) to improve coverage of plant-derived secondary metabolites [58]. Candidate annotations were ranked according to spectral similarity, mass accuracy (<2 ppm), and chemical plausibility, after which the highest-confidence assignment for each detected feature was manually inspected by comparing retention behavior and MS/MS fragmentation patterns.

Compound identification was performed by integrating MS1 and MS2 spectral information. Accurate precursor masses and isotopic distribution patterns obtained from MS1 spectra were used to assign putative molecular formulas, whereas MS2 fragmentation spectra were compared with reference spectra in mzCloud and evaluated based on diagnostic product ions and chemically plausible fragmentation pathways. Candidate identities were further assessed using mass accuracy, isotope pattern, retention behavior, spectral similarity, and database records from ChemSpider and The Natural Products Atlas. According to the Metabolomics Standards Initiative (MSI) classification, compounds supported by MS/MS spectral-library matching were reported as putatively annotated compounds (Level 2), whereas assignments based primarily on accurate mass, molecular formula, or compound-class information were classified as Level 3. Authentic reference standards were not analyzed under the same UHPLC–MS/MS conditions; therefore, none of the metabolite identities obtained from the untargeted analysis were considered confirmed Level 1 identifications. Accordingly, all reported metabolite identities from the untargeted analysis should be regarded as putative unless explicitly stated otherwise.

### 3.5. Polyphenol Profile Analysis

Polyphenolic constituents were characterized using high-performance liquid chromatography (HPLC) according to a previously reported method with minor modifications [30]. Prior to analysis, extracts were diluted with 50% (*v*/*v*) ethanol and filtered through 0.45 μm syringe filters to remove particulate material. Chromatographic analysis was performed using a Shimadzu HPLC system equipped with a photodiode array detector (CTO-20AC, Shimadzu, Kyoto, Japan). Separation was achieved on a Restek Ultra C18 reverse-phase column (250 × 4.6 mm, 5 μm) maintained at 40 °C. Detection was monitored at 280 nm. The mobile phase consisted of solvent A (95% water and 5% formic acid) and solvent B (85% acetonitrile, 10% water, and 5% formic acid). Samples were injected at a volume of 10 μL, and chromatographic separation was carried out at a constant flow rate of 1.0 mL/min. The gradient program was initiated with 20% solvent B for 4 min, followed by a linear increase to 75% B over the subsequent 8 min. This composition was maintained for 2 min before decreasing to 30% B between 14 and 17 min and finally returning to 5% B during the last minute of the run.

Eight phenolic compounds, namely gallic acid, *o*-coumaric acid, *p*-coumaric acid, chlorogenic acid, catechin, epicatechin, naringin, and quercetin, were used as reference standards for compound identification and quantification. Gallic acid, *o*-coumaric acid, and *p*-coumaric acid were purchased from Sigma-Aldrich (Fluka, St. Louis, MO, USA; purity ≥ 98%), while chlorogenic acid, catechin, epicatechin, naringin, and quercetin were obtained from Sigma-Aldrich (St. Louis, MO, USA; purity ≥ 98%). Stock solutions of each standard were prepared at a concentration of 1 mg/mL in methanol and stored at −20 °C until use. Working standard solutions at concentrations ranging from 12.5 to 100 μg/mL were prepared by two-fold serial dilution for the construction of external calibration curves. Linearity was confirmed over this range with coefficients of determination (R^2^) of 0.986, 0.999, 0.999, 0.987, 0.996, 0.988, 0.983, and 0.983 for gallic acid, *o*-coumaric acid, *p*-coumaric acid, chlorogenic acid, catechin, epicatechin, naringin, and quercetin, respectively. Each polyphenolic compound was identified by comparing its retention time and UV spectrum, acquired using the photodiode array detector, with those of the corresponding authentic reference standard. Quantification was performed using external calibration curves, and results were expressed as milligrams per gram of extract (mg/g extract).

### 3.6. Cell Culture and Cytotoxicity Assay

Four cell types were used to capture both AGA-relevant follicular biology and broader safety profiling: human hair follicle dermal papilla cells (HFDPCs; iCell Bioscience Inc., Shanghai, China), murine macrophages (RAW 264.7; ATCC TIB-71), hTERT-immortalized cells (hTERT fibroblast; ATCC CRL-4066), and human prostate cancer cells (DU-145; ATCC HTB-81). HFDPCs were maintained in a commercial dermal-papilla growth medium, RAW 264.7 and hTERT fibroblast cells were cultured in Dulbecco’s Modified Eagle Medium (DMEM), and DU-145 cells were maintained in Roswell Park Memorial Institute 1640 Medium (RPMI-1640). All media were supplemented with 10% (*v*/*v*) fetal bovine serum (FBS) and 1% (*v*/*v*) penicillin-streptomycin (100×), and cultures were maintained at 37 °C in a humidified atmosphere of 5% CO_2_.

Cytotoxicity of the UN-NE and TRI-NE extracts was screened across a ten-point, two-fold serial dilution series (0.004–2.000 mg/mL) in all four cell lines using the sulforhodamine B (SRB) colorimetric assay. Cells were seeded at 1 × 10^5^ cells/mL in 96-well plates and allowed to adhere for 24 h before a further 24 h of extract exposure. Following treatment, cells were fixed with ice-cold 50% (*w*/*v*) trichloroacetic acid for 1 h at 4 °C, stained with 0.04% (*w*/*v*) SRB solution for 30 min at room temperature, and rinsed with 1% (*v*/*v*) acetic acid to remove unbound dye; protein-bound dye was then solubilized in 10 mM Tris base (pH 10.5), and absorbance was read at 515 nm [32].

### 3.7. Paracrine-Mediated Fibroblast Proliferation Assay

HFDPCs influence the surrounding follicular microenvironment largely through secreted paracrine factors rather than direct contact, and this indirect signaling was captured using a conditioned-medium model. In this experiment, HFDPCs were seeded at 1 × 10^5^ cells/mL in a plate and incubated for 24 h. Afterwards, HFDPCs were treated with UN-NE and TRI-NE extracts or standard control (minoxidil) at a concentration of 0.0625 mg/mL for 24 h, after which the culture supernatant was collected, clarified by centrifugation (300× *g*, 3 min, 4 °C), and filtered (0.22 µm) to obtain sterile conditioned medium.

In parallel, hTERT fibroblast cells were seeded separately at 1 × 10^5^ cells/mL and incubated for 24 h. After that, the culture medium was replaced with the collected conditioned medium from treated HFDPCs. After 24 h of treatment, hTERT fibroblast cell proliferation was assessed using the SRB experiment [29].

### 3.8. K_ATP_ Channel-Dependent Viability Assay

The capacity of the UN-NE and TRI-NE extracts to sustain HFDPC viability under pharmacological K_ATP_ channel blockade, a mechanism relevant to the action of minoxidil, was evaluated using a tolbutamide (TBT)-based inhibition model. In this study, HFDPCs were seeded at 1 × 10^5^ cells/mL in a plate and incubated for 24 h. Subsequently, HFDPCs were exposed to 2.5 mM TBT for 2 h to induce channel blockade, after which the medium was replaced with UN-NE and TRI-NE extracts or minoxidil-containing medium for a further 24 h. Viability was quantified by the SRB assay and expressed relative to an untreated, non-TBT-exposed control [59].

### 3.9. Anti-Inflammatory Activity: Nitric Oxide Assay

Anti-inflammatory activity was assessed by quantifying nitrite accumulation, a stable surrogate for nitric oxide (NO) production, in LPS-stimulated RAW 264.7 macrophages and HFDPCs using a Griess reaction-based colorimetric assay [29]. Cells were seeded at 1 × 10^5^ cells/mL in a plate and incubated for 24 h. Afterward, cells were pre-treated with UN-NE and TRI-NE extracts or diclofenac sodium (reference anti-inflammatory standard) for 2 h before LPS stimulation (1 µg/mL) for a further 24 h. Nitrite concentration in the culture supernatant was interpolated from a sodium nitrite calibration curve (0.01–50 µM).

### 3.10. Lipid Peroxidation Assay

Downstream oxidative membrane damage was assessed by quantifying malondialdehyde (MDA), the principal end-product of lipid peroxidation, using the thiobarbituric acid-reactive substances (TBARS) assay [30]. In short, HFDPCs were seeded at 1 × 10^5^ cells/mL in a plate and incubated for 24 h. After that, HFDPCs were pre-treated with UN-NE and TRI-NE extracts or L-ascorbic acid for 24 h before the H_2_O_2_ challenge (100 µM, 2 h). Cells were then lysed in a reaction mixture containing 1% Triton X-100, 0.6% thiobarbituric acid, and 15% trichloroacetic acid, heated at 100 °C for 10 min, and rapidly cooled; absorbance was read at 532 nm, and MDA levels were expressed relative to the untreated control.

### 3.11. Reactive Oxygen Species Production Inhibition (H_2_DCFDA Assay)

Intracellular ROS accumulation was measured in H_2_O_2_-challenged HFDPCs using the fluorogenic probe H_2_DCFDA (commercial ROS detection kit) [60]. First, cells were seeded at 1 × 10^5^ cells/mL in a plate and incubated for 24 h. After that, cells were pre-incubated with UN-NE and TRI-NE extracts or L-ascorbic acid (reference antioxidant standard) for 30 min before exposure to H_2_O_2_ (10^−5^ M) for a further 24 h. Cells were then washed with phosphate-buffered saline (PBS), then loaded with H_2_DCFDA according to the manufacturer’s protocol, and fluorescence intensity was recorded at 485/535 nm (excitation/emission) and expressed relative to the untreated control.

### 3.12. Gene Expression Analysis

Transcriptional effects were profiled by semi-quantitative RT-PCR across three functionally distinct gene sets: the androgen metabolism genes *SRD5A1* and *SRD5A2* (assessed in both DU-145 and HFDPCs due to the high constitutive 5α-reductase activity of the former); the pro-regression mediator transforming growth factor-beta 1 (*TGFB1*); and growth-signaling/angiogenesis genes including *CTNNB1* (Wnt/β-catenin), *SHH*, *SMO*, *GLI1* (Sonic Hedgehog), and *VEGF*, which were assessed in HFDPCs only.

In this study, cells were seeded at 1 × 10^5^ cells/mL in 6-well plates and incubated for 24 h. After adherence, cells were treated for 24 h with UN-NE and TRI-NE extracts or their relevant pharmacological reference standards: dutasteride, finasteride, and minoxidil for the *SRD5A* and *TGFB1* targets; and minoxidil and purmorphamine for the Wnt/β-catenin (*CTNNB1*), Sonic Hedgehog (*SHH*, *SMO*, *GLI1*), and angiogenesis (*VEGF*) pathways. Following treatment, total RNA was extracted, followed by cDNA synthesis and PCR amplification with gene-specific primers. Band intensities were normalized to the GAPDH housekeeping gene and expressed as the fold change relative to the vehicle-treated control [54]. The primer sequences utilized for all target genes, as well as the GAPDH housekeeping gene, are documented in Supplementary Table S3.

### 3.13. Statistical Analysis

All experiments were performed in three independent biological replicates (*n* = 3), and data are expressed as mean ± standard deviation (SD). Differences among groups were evaluated by one-way analysis of variance (ANOVA) followed by Tukey’s Honestly Significant Difference (HSD) post hoc test for pairwise comparisons, with *p* < 0.05 considered statistically significant. Statistical analyses were performed using IBM SPSS Statistics, version 23.0.

## 4. Conclusions

Tri-culture fermentation with *S. cerevisiae*, *L. plantarum*, and *A. niger* produced a chemically and functionally distinct neem extract (TRI-NE) relative to the unfermented control (UN-NE). TRI-NE exhibited markedly higher total phenolic content and antioxidant capacity than UN-NE. Moreover, TRI-NE induced extensive metabolomic remodeling, characterized predominantly by metabolite accumulation and selective enrichment of quercetin despite reductions in several other quantified polyphenols. In cell-based models, TRI-NE generally outperformed UN-NE across all evaluated endpoints, enhancing paracrine-mediated fibroblast proliferation, protecting hair follicle dermal papilla cell viability under potassium-channel blockade, suppressing lipopolysaccharide-induced inflammatory nitric oxide production, and attenuating oxidative membrane damage and intracellular reactive oxygen species. At the transcriptional level, TRI-NE downregulated androgen metabolism (*SRD5A1* and *SRD5A2*) and pro-regression genes (*TGFB1*) while upregulating Wnt/β-catenin (*CTNNB1*), Sonic Hedgehog (*SHH*, *SMO*, and *GLI1*), and angiogenesis (*VEGF*)-related genes, with activity comparable to or exceeding standard hair-loss therapeutics for most targets. Collectively, these findings indicate that the superior bioactivity of TRI-NE relative to UN-NE was associated not with bulk polyphenol accumulation but rather with qualitative, fermentation-driven compositional transformation, supporting the potential of tri-culture fermentation to enhance the functional properties of neem. These findings also suggest that tri-culture fermentation may be applicable across chemically distinct botanical substrates. Nevertheless, further mechanistic studies—including dose–response evaluation to confirm the concentration dependency of the observed effects, three-dimensional (3D) in vitro hair follicle modeling, and in vivo evaluations—are required to further validate the therapeutic potential of TRI-NE as a cosmeceutical candidate for AGA.

## Figures and Tables

**Table 1:** Polyphenol composition (mg/g extract) of neem extracts determined by HPLC analysis.

Polyphenol	Extracts (mg/g Extract)	
	UN-NE	TRI-NE
Gallic acid	5.96 ± 0.00	5.95 ± 0.01
Chlorogenic acid	15.85 ± 0.12	10.86 ± 0.48
Catechin	18.55 ± 0.22	16.59 ± 0.25
Epicatechin	4.06 ± 0.11	2.27 ± 0.01
*p*-coumaric acid	2.84 ± 0.03	0.10 ± 0.03
Naringin	9.93 ± 0.52	ND
*o*-coumaric acid	0.73 ± 0.06	0.55 ± 0.02
Quercetin	10.23 ± 0.75	15.39 ± 1.97

Abbreviations: UN-NE, unfermented neem extract; TRI-NE, tri-culture fermented neem extracts.

## Data Availability

Data are contained within this article and the Supplementary Materials. Further inquiries can be directed to the corresponding author.

## Supplementary Materials

The following supporting information can be downloaded at: https://www.mdpi.com/article/10.3390/plants15182779/s1, Table S1: Cell viability (%) of HFDPCs, RAW 264.7, hTERT, and DU-145 cells treated with neem extracts via SRB assay; Table S2: List of chemicals, reagents, and reference compounds used in the study, including their purity, grades, and commercial sources; Table S3: Primer sequences of androgen metabolism genes (*SRD5A1* and *SRD5A2*), transforming growth factor-beta (*TGFB1*), Wnt/β-catenin (*CTNNB1*), Sonic Hedgehog (*SHH*, *SMO*, and *GLI1*), and angiogenesis (*VEGF*), alongside the GAPDH housekeeping gene; Table S4: Differentially abundant metabolites between the TRI-NE and UN-NE groups.
