# Supplementary material for: Tri-Culture Fermentation of Neem (Azadirachta indica) Leaves Induces Phytochemical Remodeling and Enhances Multi-Target Bioactivities Relevant to Androgenetic Alopecia
Source: Plants (Basel). 2026 Sep 10;15(18):2779. doi: 10.3390/plants15182779 (PMC13610621; doi:10.3390/plants15182779)
Supplement: Supplementary file 1 [file plants-15-02779-s001.zip › Supplementary Table S1- Cell viability.pdf]

Table S1: Cell viability (%) of HFDPCs, RAW 264.7, hTERT, and DU-145 cells treated with neem extracts via SRB assay

**RAW 264.7**		**Concentration (mg/mL)**									
		**2.000**	**1.000**	**0.500**	**0.250**	**0.125**	**0.063**	**0.031**	**0.016**	**0.008**	**0.004**
**UN-NE**	**Viability**	41.36	46.82	53.94	68.27	82.15	90.48	95.63	98.21	100.37	99.94
	**S.D.**	1.45	1.78	1.02	1.63	1.89	1.24	1.56	1.09	1.97	1.31
**TRI-NE**	**Viability**	38.72	44.15	61.68	79.93	81.47	89.36	94.82	97.65	99.83	100.42
	**S.D.**	1.67	1.13	1.85	1.28	2.01	1.46	1.09	1.72	1.38	1.95
**Diclofenac sodium**	**Viability**	42.18	51.35	64.82	82.4	89.26	95.15	99.08	98.74	100.12	42.18
	**S.D.**	1.52	1.34	1.67	1.18	1.92	1.05	1.43	1.21	1.86	1.52

**HFDPC**		**Concentration (mg/mL)**									
		**2.000**	**1.000**	**0.500**	**0.250**	**0.125**	**0.063**	**0.031**	**0.016**	**0.008**	**0.004**
**UN-NE**	**Viability**	35.47	42.63	49.85	58.32	71.94	84.28	92.56	97.13	99.47	100.28
	**S.D.**	1.58	1.23	1.91	1.06	1.74	1.37	1.62	1.09	1.85	1.48
**TRI-NE**	**Viability**	33.92	40.18	47.63	56.87	80.35	83.19	91.84	96.72	99.15	100.53
	**S.D.**	1.71	1.05	1.48	1.93	1.16	1.67	1.32	2.04	1.09	1.86
**Dutasteride**	**Viability**	29.12	37.45	49.80	63.12	72.45	86.28	88.92	96.15	98.70	29.12
	**S.D.**	1.45	1.62	1.28	1.85	1.30	1.64	1.21	1.50	1.18	1.45
**Finasteride**	**Viability**	27.65	35.80	47.95	61.40	70.85	84.82	89.15	95.30	99.10	27.65
	**S.D.**	1.82	1.15	1.74	1.38	1.95	1.22	1.56	1.08	1.42	1.82
**Minoxidil**	**Viability**	33.85	42.90	54.75	67.80	80.18	88.35	94.20	97.65	99.82	33.85
	**S.D.**	1.32	1.78	1.50	1.12	1.65	1.40	1.88	1.15	1.25	1.32
**Purmorphamine**	**Viability**	30.50	38.25	51.10	64.30	73.15	89.45	97.10	96.80	100.05	30.50
	**S.D.**	1.60	1.40	1.85	1.20	1.48	1.75	1.10	1.35	1.62	1.60

**hTERT**		**Concentration (mg/mL)**									
		**2.000**	**1.000**	**0.500**	**0.250**	**0.125**	**0.063**	**0.031**	**0.016**	**0.008**	**0.004**
**UN-NE**	**Viability**	37.85	44.29	51.73	61.48	73.62	85.94	93.28	97.85	99.62	100.14
	**S.D.**	1.63	1.28	1.94	1.11	1.57	1.82	1.36	1.09	1.98	1.45
**TRI-NE**	**Viability**	36.14	42.87	50.16	59.73	82.48	84.65	92.37	97.02	99.31	100.47
	**S.D.**	1.19	1.86	1.53	1.02	1.77	1.34	1.91	1.08	1.65	1.42
**Minoxidil**	**Viability**	35.42	42.18	56.3	69.15	80.24	88.65	94.12	97.8	99.45	35.42
	**S.D.**	1.82	1.45	1.28	1.74	1.56	1.12	1.39	1.08	1.65	1.82

**DU-145**		**Concentration (mg/mL)**									
		**2.000**	**1.000**	**0.500**	**0.250**	**0.125**	**0.063**	**0.031**	**0.016**	**0.008**	**0.004**
**UN-NE**	**Viability**	43.28	48.65	56.37	70.94	83.62	91.75	96.28	99.14	100.53	99.87
	**S.D.**	1.52	1.86	1.19	1.71	1.04	1.63	1.38	1.92	1.27	1.58
**TRI-NE**	**Viability**	40.15	45.83	54.29	80.16	82.94	90.87	95.74	98.63	100.19	100.61
	**S.D.**	1.34	1.79	1.06	1.95	1.42	1.15	1.83	1.29	1.68	1.01
**Dutasteride**	**Viability**	28.45	36.12	48.70	62.35	71.80	80.15	89.40	95.82	98.90	28.45
	**S.D.**	1.65	1.42	1.88	1.25	1.90	1.48	1.15	1.72	1.30	1.65
**Finasteride**	**Viability**	34.18	43.65	55.40	68.90	80.12	88.75	94.60	97.85	99.50	34.18
	**S.D.**	1.55	1.28	1.74	1.60	1.35	1.82	1.09	1.45	1.20	1.55
**Minoxidil**	**Viability**	40.25	49.80	63.15	80.35	85.90	91.80	95.95	98.40	100.15	40.25
	**S.D.**	1.40	1.75	1.18	1.52	1.68	1.22	1.50	1.10	1.78	1.40
