# Supplementary material for: Tri-Culture Fermentation of Neem (Azadirachta indica) Leaves Induces Phytochemical Remodeling and Enhances Multi-Target Bioactivities Relevant to Androgenetic Alopecia
Source: Plants (Basel). 2026 Sep 10;15(18):2779. doi: 10.3390/plants15182779 (PMC13610621; doi:10.3390/plants15182779)
Supplement: Supplementary file 1 [file plants-15-02779-s001.zip › Supplementary Table S2-List of chemicals.pdf]

Table S2: List of chemicals, reagents, and reference compounds used in the study, including their purity, grades, and commercial sources.

Category	Item	Source
Standard compounds	- Epigallocatechin gallate (EGCG)<br>- Gallic acid<br>- Trolox (6-hydroxy-2,5,7,8-tetramethylchroman-2-carboxylic acid)<br>- Diclofenac sodium<br>- L-ascorbic acid<br>- 2,2-diphenyl-1-picrylhydrazyl (DPPH)<br>- 2,2′-azino-bis(3-ethylbenzothiazoline-6-sulfonic acid) (ABTS)<br>- Sulforhodamine B (SRB)<br>- Lipopolysaccharide (LPS)<br>- Dimethyl sulfoxide (DMSO)	Sigma-Aldrich (St. Louis, MO, USA)
Hair Regeneration Standard compounds	- Dutasteride<br>- Finasteride<br>- Minoxidil<br>- Purmorphamine	Wuhan W&Z Biotech (Wuhan, China)
Chemical Reagents	- Folin-Ciocalteu reagent<br>- Aluminum chloride hexahydrate<br>- Hydrogen peroxide (H_2O_2)<br>- Trichloroacetic acid (TCA)<br>- Triton X-100	Merck (Darmstadt, Germany)
	- Thiobarbituric acid (TBA)	VWR Chemicals (BDH Chem. Ltd., Poole, UK)
Cell Culture & Assays	- Dulbecco's Modified Eagle Medium (DMEM)<br>- Roswell Park Memorial Institute 1640 Medium (RPMI-1640)<br>- Fetal bovine serum (FBS)<br>- Penicillin-streptomycin solution (100 ×)	Gibco Life Technologies<br>(Thermo Fisher Scientific, Waltham, MA, USA)
	- Fibroblast Cell Growth Medium	Promo Applied Biological Materials Inc. (Richmond, Canada)
	- Griess reagent colorimetric assay kit	Invitrogen (Thermo Fisher Scientific, Inc., Eugene, OR, USA)
Cell Lines	- Human hair follicle dermal papilla cells (HFDPCs; catalog no. 2502)	iCell Bioscience Inc. (Shanghai, China)
	- Murine macrophage cells (RAW 264.7; ATCC TIB-71)<br>- Human prostate cancer cells (DU-145; ATCC HTB-81)<br>- Human dermal fibroblast cells (hTERT; ATCC CRL-4066)	American Type Culture Collection (Rockville, MD, USA)
Software	- LabSolutions version 1.86	Shimadzu (Kyoto, Japan)
	- ImageJ version 1.53t	NIH (Bethesda, MD, USA)
	- Image Lab™ version 5.1	Bio-Rad Laboratories (Hercules, CA, USA)
