# Supplementary material for: Tri-Culture Fermentation of Neem (Azadirachta indica) Leaves Induces Phytochemical Remodeling and Enhances Multi-Target Bioactivities Relevant to Androgenetic Alopecia
Source: Plants (Basel). 2026 Sep 10;15(18):2779. doi: 10.3390/plants15182779 (PMC13610621; doi:10.3390/plants15182779)
Supplement: Supplementary file 1 [file plants-15-02779-s001.zip › Supplementary Table S3- Primer sequences .pdf]

Table S3: Primer sequences of androgen metabolism genes (*SRD5A1* and *SRD5A2*), transforming growth factor-beta (*TGFB1*), Wnt/β-catenin (*CTNNB1*), Sonic Hedgehog (*SHH*, *SMO*, and *GLI1*), and angiogenesis (*VEGF*), alongside the *GAPDH* housekeeping gene.

Target Category	Biological Pathway	Target Gene	Accession Number	Forward Sequence	Reverse Sequence
Hair loss factor	5α-reductase	*SRD5A1*	NM_001047.4	AGCCATTGTGCAGTGTATGC	AGCCTCCCCTTGGTATTTTG
		SRD5A2	NM_000348.4	TGAATACCCTGATGGGTGG	CAAGCCACCTTGTGGAATC
		SRD5A3	NM_024592.5	TCCTTCTTTGCCCAAACATC	TCCTTCTTTGCCCAAACATC
	Transforming growth factor beta 1	*TGFB1*	NM_00600.7	AACCCACAACGAAATCTATG	CTTTTAACTTGAGCCTCAGC
Hair regrowth stimulation	Sonic Hedgehog	*SHH*	NM_000193.4	AAAAGCTGACCCCTTTAGCC	GCTCCGGTGTTTTCTTCATC
		SMO	NM_005631.5	GAAGTGCCCTTGGTTCGGACA	CCGCCAGTCAGCCACGAAT
		GLI1	NM_005269.3	GCAGGGAGTGCAGCCAATACAG	GAGCGGCGGCTGACAGTATA
	Wnt/β-catenin	*CTNNB1*	NM_001330729.2	CCCACTAATGTCCAGCGTTT	AACCAAGCATTTTCACCAGG
	Angiogenesis	*VEGF*	NM_001025366.3	CTACCTCCACCATGCCAAGT	GCGAGTCTGTGTTTTTGCAG
Internal control		*GAPDH*	NM_001289745.3	GGAAGGTGAAGGTCGGAGTC	CTCAGCCTTGACGGTGCCATG
